# A Biomimetic Lubricant Captures Hyaluronic Acid In Situ to Regenerate Cartilage: From Bench to Bedside

**DOI:** 10.1002/advs.202600049

**Published:** 2026-04-10

**Authors:** Yongan Lin, Zijian Yan, Jiayi Chen, Xiaochao Wang, Ruibin Lin, Yunqi Fu, Zhaoying Lv, Bohui Wu, Xuewei Cao, Renjian Xie, Ming Dong, Chenxiao Zheng, Li Ren

**Affiliations:** ^1^ School of Materials Science and Engineering South China University of Technology Guangzhou China; ^2^ National Engineering Research Center for Tissue Restoration and Reconstruction Guangzhou China; ^3^ Guangzhou National Laboratory Guangzhou International BioIsland Guangzhou China; ^4^ Guangzhou Proud Seeing Biotechnology Co., Ltd. Guangzhou China; ^5^ Zhongshan Hospital of Traditional Chinese Medicine Affiliated to Guangzhou University of Traditional Chinese Medicine Zhongshan China; ^6^ Orthopaedic Hospital of Guangdong Provincial Hospital of Traditional Chinese Medicine Guangzhou China; ^7^ School of Medical Information Engineering Gannan Medical University Ganzhou China; ^8^ State Key Laboratory of Respiratory Disease The First Affiliated Hospital of Guangzhou Medical University Guangzhou Medical University Guangzhou China

**Keywords:** first‐in‐human pretrial, hyaluronic acid, osteoarthritis, self‐assembly, two‐step injection

## Abstract

Lubrication deficiency can lead to cartilage degeneration and osteoarthritis (OA), for which hyaluronic acid (HA) is limited by its short retention and reparative effect. Inspired by the natural cartilage lubrication mechanism, we propose a two‐step injection strategy to construct a biomimetic synergistic lubrication coating in situ on cartilage surfaces. The administration of developed cationic biomimetic lubricant (CS‐g‐PM) as the first injection to capture the subsequently administered HA via in situ self‐assembly on the cartilage surface, forming a natural cartilage‐like lubrication layer. In vitro, this strategy reduced friction coefficients to normal cartilage levels in OA human cartilage. Moreover, sequential CS‐g‐PM and HA articular‐injection in OA rats extended HA retention 5‐fold versus HA alone; meanwhile, regeneration of cartilage was synergistically promoted. Importantly, a first‐in‐human pretrial demonstrated rapid MRI‐evident cartilage repair at 1 month. At 6 months of follow‐up, patients showed sustained functional improvement and halted structural degeneration. This strategy offers a transformative solution for OA treatment.

## Graphical abstract

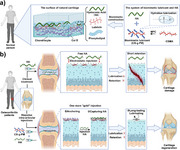

## 1 Introduction

Osteoarthritis (OA), a prevalent degenerative joint disease affecting over 500 million individuals worldwide [1], is characterized by progressive cartilage degradation and impaired joint lubrication [2, 3]. Increasing research affirms that impairment of cartilage lubrication is a primary factor contributing to OA [4, 5, 6]. Current clinical management often relies on intra‐articular HA injection to restore synovial fluid viscoelasticity [7, 8]. However, due to the poor electrostatic interactions between negatively charged HA and cartilage surfaces with the densely negatively charged [9, 10], HA exhibits poor adhesion and rapid clearance, leading to transient symptom relief without structural repair [9, 11, 12]. In healthy joints, articular cartilage achieves extremely low friction through a natural lubrication complex composed of HA, lubricin, and phospholipids, which forms a stable boundary layer on the cartilage surface. This layer is anchored via lubricin, while phospholipids facilitate hydration lubrication under load [13–15]. In OA, this native interface is disrupted, resulting in increased friction and accelerated wear [16–18]. Therefore, capturing and enriching HA on the cartilage surface and restoring a functional lubrication interface, perhaps, could represent a promising therapeutic strategy for halting OA progression.

Recent attempts to enhance HA retention have focused on cartilage surface modification [19] or HA functionalization with targeting moieties [6, 20, 21]. For example, cartilage modification using HA‐binding peptides has shown promise in studies, extending HA retention and improving lubrication in vitro [19]. While these approaches show preclinical promise, they often fail to recapitulate the synergistic, multi‐component nature of natural lubrication. Similarly, HA functionalization with collagen type II (Col II) binding peptides or chemical groups (e.g., catechol, choline phosphate, sulfonates, etc.) for covalent or non‐covalent binding to Col II has demonstrated enhanced HA retention in animal models, while chemical modification of HA may compromise its bioactivity and biocompatibility. Therefore, a biomimetic strategy that reconstructs the native lubrication interface without altering HA structure remains highly desirable.

**SCHEME 1:**
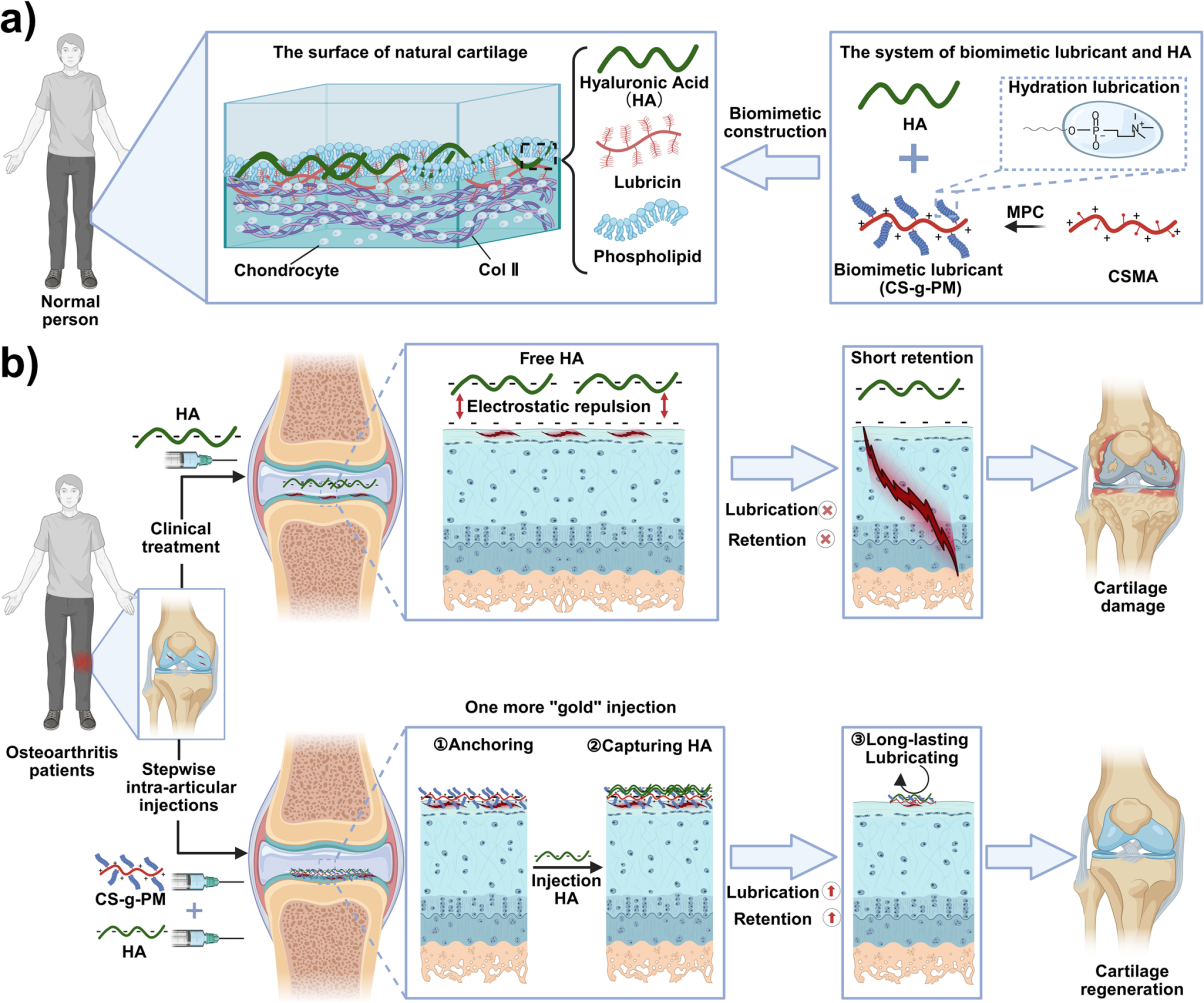
Illustration of the overall idea. (a) The outstanding lubricative properties of natural articular cartilage arise from a composite coating composed of HA, lubricin, and phospholipids at its surface. Notably, lubricin facilitates the enrichment of synovial HA onto cartilage surfaces, and HA complexes with phospholipids, a key component to maintain extremely low friction under high pressure via hydration lubrication. In this way, articular cartilage is conferred exceptional lubrication characteristics. Here, a biomimetic brush‐like cationic polyelectrolyte (CS‐g‐PM) was designed through grafting phosphorylcholine‐polymer onto a chitosan backbone as side chains, thereby mimicking the functionality of natural lubricin and phospholipids. (b) Current clinical management of OA predominantly relies on intra‐articular HA injections. However, the inherent negative charge of cartilage surfaces severely limits HA binding, with administered HA in a free state after articular injection, which results in limited joint residence time, ineffective lubrication, and ultimately controversial clinical outcomes without preventing cartilage degradation. To address these limitations, we developed a two‐step injection strategy, where we employed CS‐g‐PM as the first injection to anchor on the articular cartilage surface, followed by capturing HA. The cationic CS‐g‐PM adsorbs and assembles with HA to enable enrichment of HA on the cartilage surface, and simultaneously forms a mimic lubricating complex to restore remarkable lubrication. Thus, the limitation of a single injection of HA for OA treatment in the clinic could be evaded by adding CS‐g‐PM as the first injection.

Inspired by the “anchoring‐capturing‐lubricating” function of lubricin, we propose an interface‐bionic synergistic lubrication coating strategy [22–24]. We designed a cationic biomimetic lubricant, CS‐g‐PM, by grafting poly(methacryloyloxyethyl phosphorylcholine) (PM) onto chitosan (CS). CS provides positive charges for cartilage adhesion [25–27], while PM mimics phospholipids to enhance hydration lubrication. Although recent advances in cationic or zwitterionic chitosan copolymers have demonstrated their potential as effective boundary lubricants [28, 29], we herein report a transformative two‐step strategy that moves beyond lubrication replacement to enable HA‐binding cartilage regeneration. Through stepwise intra‐articular injection, CS‐g‐PM first adsorbs onto cartilage, followed by capturing HA, enabling in situ electrostatic self‐assembly into a composite lubrication coating that mimics the natural interface. Herein, we demonstrate that this coating not only restores boundary lubrication but also synergistically enhances HA retention and promotes cartilage regeneration. We validate its tribological performance on human OA cartilage, its retention and reparative effects in rat OA models, and its safety and Potential efficacy in a first‐in‐human pilot trial. This work establishes a clinically feasible two‐step strategy to overcome the limitations of HA therapy, offering a new pathway toward OA treatment.(Scheme 1)

## 2 Results

### 2.1 Synthesis of CS‐g‐PM and Construction of Lubricating Coatings for Articular Cartilage

**FIGURE 1:**
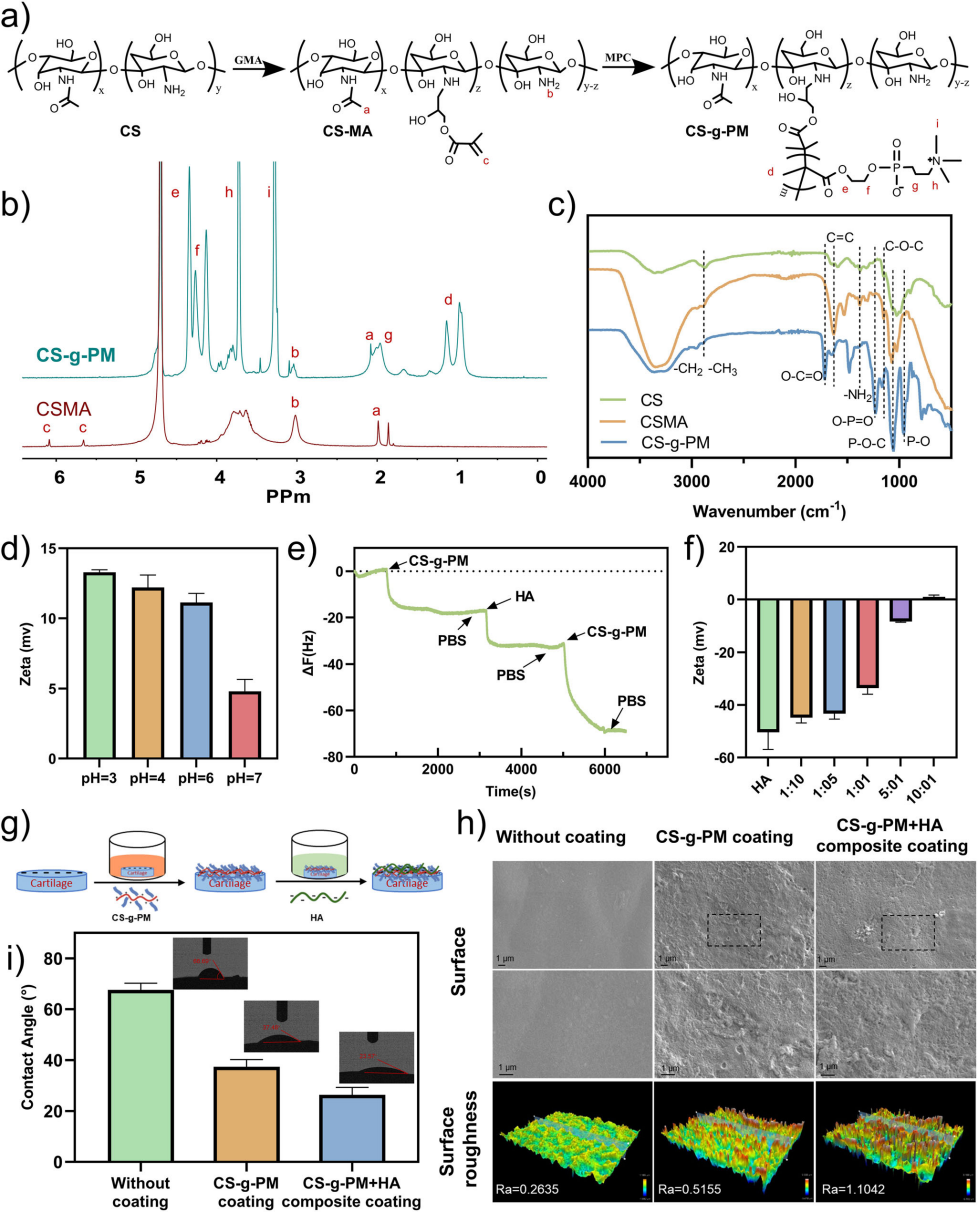
Synthesis of CS‐g‐PM and construction of lubricating coatings for articular cartilage. (a) Synthesis roadmap of CS‐g‐PM. (b) ^1^H NMR spectra and molecular structure of CSMA and CS‐g‐PM. (c) The FTIR spectra of CS, CSMA, and CS‐g‐PM. (d) The Zeta potential of CS‐g‐PM at different pH levels. (e) QCM‐D absorption curves revealed the interaction between CS‐g‐PM and HA. (f) The zeta potential of CS‐g‐PM and HA complexes with different mass ratios. (g) Schematic diagram of HA coating construction on cartilage surface. (h) The micromorphology and surface roughness of the samples without coating, CS‐g‐PM coating, and CS‐g‐PM+HA composite coating. (i) The water contact angle of without coating, CS‐g‐PM coating, and CS‐g‐PM+HA composite coating.

Inspired by the lubricin structure and function of native cartilage, we designed a cationic biomimetic lubricant coating system to facilitate HA enrichment on cartilage surfaces. Chitosan is the most abundant cationic polyelectrolyte in nature and has exhibited wide applications as a biomaterial in the treatment of OA [30]. It is well known that zwitterionic PM polymer with phosphorylcholine (PC) headgroups provides highly efficient lubrication [31]. In this work, brush‐like cationic biomimetic lubricants (CS‐g‐PM) were synthesized using the “grafting from” method, and the double bond modified CS chain acts as a macroinitiator to induce graft polymerization of 2‐Methacryloyloxyethyl phosphorylcholine (MPC) monomers (Figure 1a). The resultant CS‐g‐PM exhibited improved aqueous solubility compared to native chitosan, dissolving completely in deionized water, PBS (pH 7.4), and cell culture media (Figure S1). This solubility enhancement, attributed to the hydrophilic PC moieties, is critical for intra‐articular injection applications. Structural confirmation was performed via ^1^H‐NMR and FTIR spectroscopy. In the ^1^H‐NMR spectrum (Figure 1b), the peaks at 5.65 and 6.08 ppm are assigned to signals of alkene hydrogen, proving the successful synthesis of double bond modified chitosan (CSMA), and further calculating the degree of double bond substitution of 30% based on the ratio of hydrogen on the methyl group to the alkene peak area. The characteristic peaks of chitosan (2.08–4.01 ppm) coexisted with PMPC proton signals (0.96–4.34 ppm), while the disappearance of alkene hydrogen signals (5.65–6.08 ppm) confirmed successful copolymerization. Therefore, the grafting rate of PM can be considered to be approximately 30%. FTIR analysis (Figure 1c) revealed new absorption bands at 1720 cm^−1^ (C═O), 1229 cm^−1^ (O─P═O), and 1049 cm^−1^ (P─O─C), indicative of PC moiety grafting, alongside chitosan backbone vibrations (1633 cm^−1^ for C═C, 1150 cm^−1^ for C─O─C). In addition, the zeta potential of CS‐g‐PM is tested at different pH values, and the results are shown in Figure 1d. CS‐g‐PM is positively charged and decreases with increasing pH due to the amino group on CS protonation. To directly visualize and quantify this brush‐like morphology, we performed atomic force microscopy (AFM) on CSMA and CS‐g‐PM samples (Figure S2). The AFM height images reveal a striking morphological transformation. CSMA exhibits a smooth and homogeneous surface, with a Ra of 0.279 nm. CS‐g‐PM displays a pronounced “brush‐like” characteristic characterized by numerous nanoscale protrusions. This corresponds to a dramatic increase in surface roughness, with the Ra value rising to 3.82 nm. The observed surface roughness and nanoscale protrusions are consistent with the expected architecture of a graft copolymer with flexible side chains extending from a polysaccharide backbone.

To demonstrate the feasibility of CS‐g‐PM to adsorb and capture HA to restore the lubricating complex in cartilage, the interaction of CS‐g‐PM with HA was first characterized by Quartz crystal microbalance (QCM). The analysis (Figure 1e) demonstrated rapid electrostatic adsorption of CS‐g‐PM onto negatively charged surfaces (Δf = −18 Hz), followed by HA binding (Δf = −31 Hz) and a secondary CS‐g‐PM injection causing further frequency decreases (Δf = −68 Hz), confirming stepwise layer‐by‐layer assembly. The zeta potential also reflects the interaction between CS‐g‐PM and HA. As shown in Figure 1f, the zeta potential of the CS‐g‐PM and HA complexes changed from negative to positive as the concentration of CS‐g‐PM increased. These results indicate the affinity of CS‐g‐PM with HA to form a coating on the surface.

Furthermore, as illustrated in Figure 1g, cartilage samples were used to check the surface coating formed by electrostatic self‐assembly of CS‐g‐PM and HA. First, the surface morphology of cartilage samples was observed by SEM and 3D profilometer, and the results are shown in Figure 1h. The surface of articular cartilage was relatively smooth and flat, whereas the surface of the cartilage became rough after soaking in CS‐g‐PM or soaking in CS‐g‐PM followed by soaking in HA, and its surface roughness was 0.2635, 0.5155, and 1.1042, respectively, which indicated the successful construction of the CS‐g‐PM or CS‐g‐PM+HA coating. Further, the hydrophilicity of the coating was evaluated by measuring the static water contact angle (WCA). In Figure 1i, the WCA value is 66.69°for bare cartilage, and becomes more hydrophilic after coating with CS‐g‐PM, while the composite coating of CS‐g‐PM+HA had better hydrophilicity. This is because the zwitterionic phosphorylcholine headgroups can bind a large number of water molecules via strong hydration, forming a hydration layer that is responsible for the excellent lubrication. CS‐g‐PM and CS‐g‐PM+HA coatings were confirmed by FTIR spectroscopy. Compared with the uncoated cartilage surface, CS‐g‐PM or CS‐g‐PM+HA coatings showed new characteristic absorption peaks at 2900, 1700, 1235, and 1036 cm^−1^, representing ─CH_2_/─CH_3_, C═O, and O─P═O, respectively (Figure S3). In addition, the XPS spectra of the CS‐g‐PM or CS‐g‐PM+HA coatings on cartilage, when compared with uncoated cartilage, exhibited a significant increase in phosphorus content. Moreover, the C/O ratio of CS‐g‐PM+HA coatings approached that of HA, indicating successful adsorption of HA onto the cartilage surface. (Figure S4 and Table S1).

### 2.2 Friction Properties of Human OA Cartilage In Vitro

**FIGURE 2:**
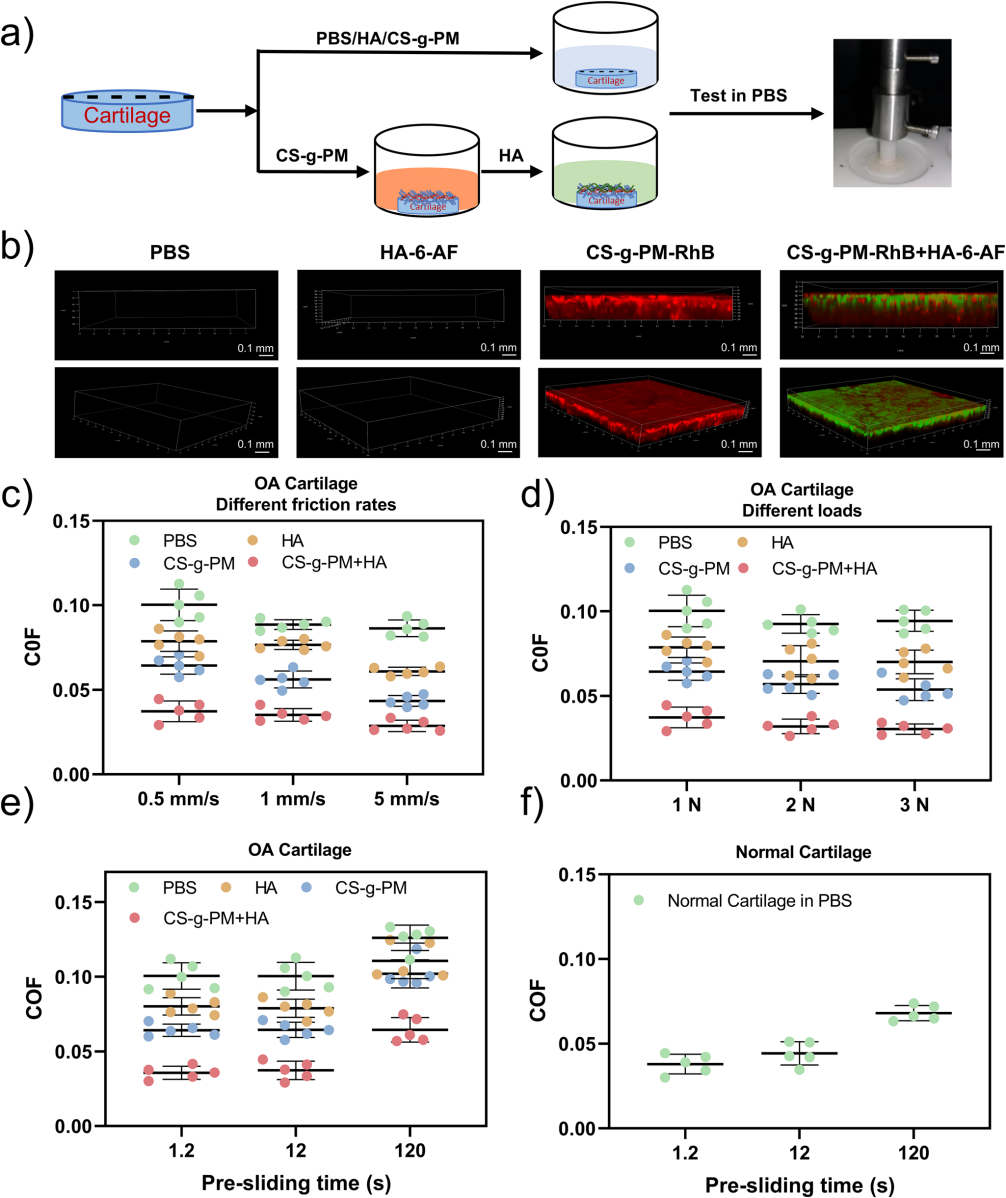
Tribology test for OA human cartilage with or without lubrication coatings (*n* = 5, the values are shown as mean ± standard deviation (SD)). (a) Schematic diagram of sample preparation and tribological test. (b) Visualization of CS‐g‐PM and HA coatings formation above human cartilage. (c) The lubrication properties of the different OA human cartilage at sliding velocities from 0.5 to 5.0 mm/s under loads from 1 N (P≈0.05 MPa). (d) The lubrication properties of the different OA human cartilage at sliding velocities from 0.5 mm/s under loads from 1 to 3 N (P≈0.05‐0.1 MPa). (e) The COF of different OA human cartilage following pre‐sliding treatment under load for different durations. (f) The COF of normal human cartilage following pre‐sliding treatment under load for different durations.

Using a previously reported method [6], isolated human cartilage was treated with trypsin to form a sample mimicking OA cartilage. In Figure S5, Normal human cartilage surfaces are flat and smooth, whereas mimicking OA‐diseased cartilage surfaces show significant wear and less cartilage matrix (lighter in color). To further explore the formation of HA coatings in human cartilage, we used confocal microscopy to visualize the coating formation process by labeling CS‐g‐PM and HA with different fluorescent molecules. The OA human cartilage sample was incubated with PBS, HA labeled with 6‐Aminofluorescein (HA‐6‐AF), CS‐g‐PM labeled with Rhodamine B (CS‐g‐PM‐RhB), or CS‐g‐PM‐RhB+HA‐6‐AF (incubated successively) at 37°C in the presence of synovial fluid proteins (human serum albumin (HSA) and γ‐globulin). Confocal imaging (Figure 2b) showed that HA did not bind the cartilage surface, due to the cartilage surface exhibiting high negative electrical characteristics, which hindered the adsorption of HA, consistent with previous studies. By contrast, positively charged CS‐g‐PM easily absorbs onto cartilage surfaces via electrostatic interactions. What's more, the successful adsorption of HA onto the cartilage surface after adsorbing the CS‐g‐PM coatings indicates that CS‐g‐PM may enhance the binding of HA on the cartilage surface. Therefore, the composite coating of CS‐g‐PM+HA can be constructed by electrostatic self‐assembly.

Figure 2a shows the actual and schematic diagrams of the tribometer process. To more accurately reflect the lubrication properties of the coatings, different human cartilage samples were tested in PBS by the universal mechanical tester (UMT). Figure 2c,d depicts the coefficients of friction (COF) of OA cartilage under varying sliding velocities or loads. The COF values of each group remain unchanged with variations in load and sliding velocity, which is consistent with the boundary lubrication regime described by the classical tribological theory of the Stribeck curve. Notably, there was no significant difference in COF between the PBS group and the HA‐treated group, indicating that HA treatment exerts a limited effect on improving lubrication performance. In contrast, the COF of the CS‐g‐PM‐treated group was significantly reduced. More interestingly, the OA cartilage treated with CS‐g‐PM+HA exhibited a further significant decrease in COF, outperforming both the HA‐only and CS‐g‐PM‐only treatment groups, suggesting that the composite coating of CS‐g‐PM and HA has a synergistic lubricating effect. Critically, under different loads and sliding velocities, the composite coating of CS‐g‐PM and HA maintained excellent lubricating performance, indicating good binding stability between the coating and the cartilage surface. Furthermore, we evaluated the COF of OA and normal cartilage following pre‐sliding treatment under load for different durations (Figure 2e,f). After treatment with different lubricants, the COF of OA cartilage gradually increased with increasing pre‐sliding duration. At the same pre‐sliding time, the OA cartilage treated with CS‐g‐PM+HA still showed the optimal lubrication performance, and its COF value was close to that of normal cartilage (Figure 2f). In Figure S6, CS‐g‐PM coating or CS‐g‐PM+HA composite coating modification similarly reduced the friction coefficient of normal cartilage. However, the reduction in the COF of OA cartilage is more significant. These findings collectively demonstrate that the CS‐g‐PM effectively binds HA to the cartilage surface, significantly enhancing HA's lubricating efficacy. Consequently, the CS‐g‐PM+HA combination effectively restores the lubrication function of diseased human cartilage. This restoration is attributed to the composite's capacity to form a stable lubricating layer on the cartilage surface.

According to the lubrication theory [32], the CS‐g‐PM+HA composite coating has excellent lubrication performance mainly due to two aspects. On the one hand. The phosphocholine group of CS‐g‐PM is zwitterionic and comprises both positively and negatively charged groups (N^+^(CH_3_)_3_) and PO_4_^3−^. These functional groups can be highly hydrated, providing excellent hydration and lubrication of cartilage [33]. On the other hand, it has been demonstrated that HA, when bound to the cartilage surface, can greatly improve the lubricating properties of cartilage, mainly attributed to the formation of a boundary lubrication layer by HA on the cartilage surface [21]. Therefore, the lubrication performance of the CS‐g‐PM+HA composite coating is derived from the synergistic effect of hydration lubrication and boundary lubrication.

### 2.3 Evaluation of Biocompatibility In Vitro and Vivo

**FIGURE 3:**
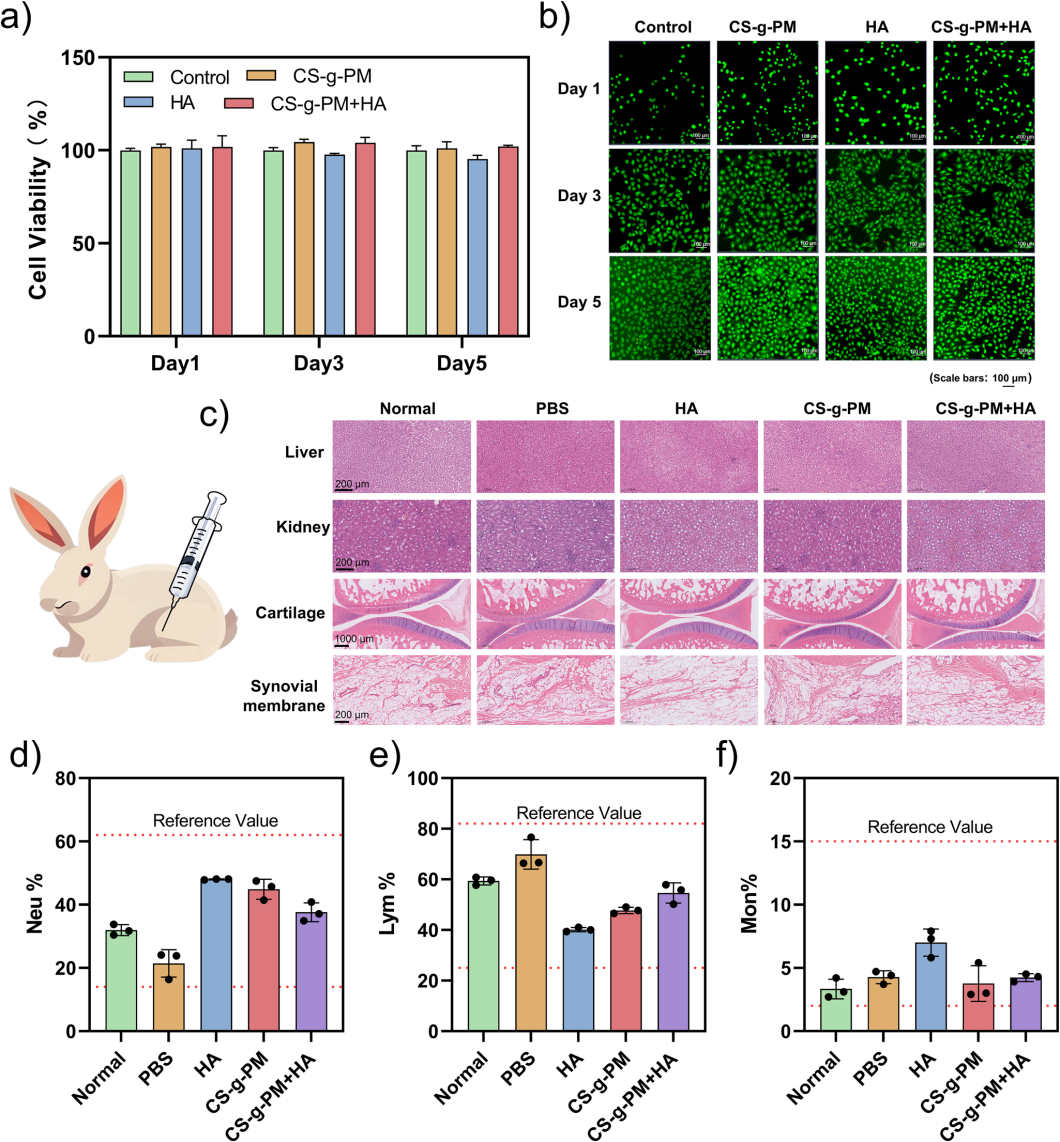
In vivo and in vitro biological safety. (a) Cell viability incubated with HA, CS‐g‐PM, or CS‐g‐PM+HA for 1, 3, and 5 days (*n* = 3). (b) Representative fluorescence images showing live (green)/dead (red) staining assay of chondrocyte co‐cultured with HA, CS‐g‐PM, or CS‐g‐PM+HA on days 1, 3, and 5. Scale bar 100 µm. (c) Representative images of cartilage, liver, and kidney tissue sections after intra‐articular injection of HA, CS‐g‐PM, or CS‐g‐PM+HA in New Zealand rabbits. (d, e) Routine blood analyses in New Zealand rabbits after intra‐articular injection of HA, CS‐g‐PM, or CS‐g‐PM+HA (*n* = 3).

As an appropriate bio‐lubricant, in addition to having excellent lubrication properties, the CS‐g‐PM should also be biocompatible. Thus, a cell counting kit‐8 (CCK‐8) assay and Live/Dead assay were used to evaluate the viability and proliferation of the C‐28/I2 (RRID:CVCL_0187; CLOUD‐CLONE CORP. WUHAN, China, Catalogue No. CSI347Hu11, Free from HIV‐1, HBV, HCV, mycoplasma, bacteria, yeast, and fungi). As seen from Figure 3a, the cell viability remained nearly 100% for CS‐g‐PM or CS‐g‐PM+HA, and no significant difference was observed between the control group at 1, 3, and 5 days, indicating the nontoxicity of CS‐g‐PM. Furthermore, the Live/Dead staining assay revealed that the C28/I2 cells increased with the culture time from day 1 to day 5, and almost no dead cells were observed at different time points (Figure 3b). Overall, these findings indicate that CS‐g‐PM and CS‐g‐PM+HA are compatible with C28/I2 cells. Lubricants injected into the joint cavity are invasive and prone to bacterial infections; therefore, the lubricant must have certain antimicrobial properties. Research indicates that chitosan exhibits its strongest activity on bacterial cell surfaces, leading to membrane permeabilization [34]. This interaction is primarily attributed to electrostatic forces arising from the positive charge of protonated amino groups in chitosan interacting with negatively charged molecules on the cell surface [35]. Typically, this increased permeability results in the leakage of intracellular substances, ultimately causing cell death [36, 37]. Therefore, we tested the antimicrobial performance of different concentrations of CS‐g‐PM against Escherichia coli and Staphylococcus aureus, as shown in Figure S7. The antimicrobial performance of CS‐g‐PM against Escherichia coli and Staphylococcus aureus was gradually enhanced with the increase of the concentration, in which the bactericidal rate of CS‐g‐PM above 1 mg/mL reached 99%.

For human clinical trials, we carried out a series of in vitro biocompatibility tests following the regulation of International Standard Organization (ISO), including the Hemolysis test, direct contact, Cytotoxicity test, Skin sensitivity test, Animal intradermal reaction test, and Acute systemic toxicity test. The results are summarized in Table S2. All the tests confirm that CS‐g‐PM met the biocompatibility requirements of ISO for medical devices. Further, we selected New Zealand rabbits for intra‐articular injection of HA or CS‐g‐PM or CS‐g‐PM+HA and evaluated the safety through cartilage histological sections and liver and kidney histological sections. The representative image of histological evaluation is shown in Figure 3c. The HE staining results of each group showed that the articular cartilage structure was intact after intra‐articular injection, and no fibrosis, matrix defect, inflammatory cell adhesion, chondrocyte necrosis, or other abnormal phenomena were found on the cartilage surface. There was also no inflammatory cell infiltration in the synovial tissue. Further histological evaluation of the liver and kidney tissues of rabbits after intra‐articular injection showed that the renal tubules and renal capsule cavities were clear and identifiable, with no inflammatory cell infiltration, no deformation, edema, or other abnormal conditions of hepatocytes and hepatic lobules. It shows that the injection of the material does not stimulate the inflammatory response of liver and kidney tissue. Subsequently, we conducted hematological examinations on rabbit whole blood, with the results presented in Figure 3d–f. The counts of inflammation‐associated cells, including centrocytes, lymphocytes, and monocytes, were found to be within the normal physiological range. Notably, no signs of an inflammatory response were observed after the injection. In summary, the combination of in vitro and in vivo evaluation shows that CS‐g‐PM has good biocompatibility.

### 2.4 In Vivo Retention Time

**FIGURE 4:**
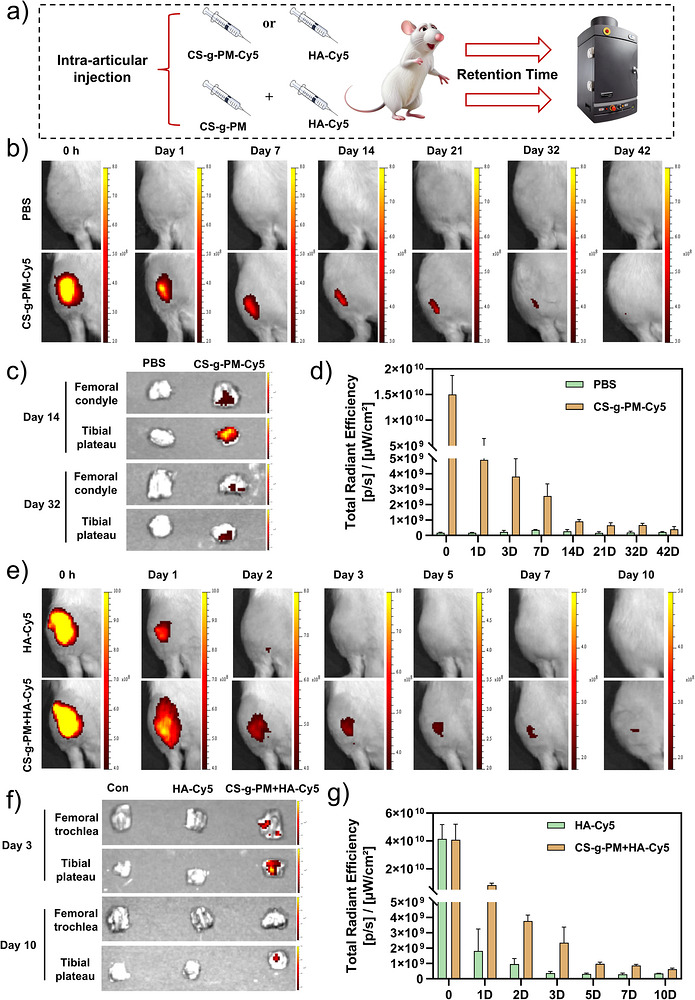
Retention time of Cy5‐labelled CS‐g‐PM and HA in the rat joint cavity. (a) Schematic diagram of the rat in vivo imaging experiment. (b) Representative fluorescence imaging with the IVIS Spectrum imaging system of rat knee joints over 42 days after intra‐articular injection of PBS or CS‐g‐PM‐Cy5. (*n* = 3). (c) The fluorescence signals of CS‐g‐PM‐Cy5 adhesion on the rat femoral condyle or tibia platform on days 14 and 32. (d) Quantitative analysis of the fluorescence intensity of PBS and CS‐g‐PM‐Cy5 group. (e) Representative fluorescence imaging with the IVIS Spectrum imaging system of rat knee joints over 10 days after intra‐articular injection of HA‐Cy5 or CS‐g‐PM+HA‐Cy5. (*n* = 3). (f) The fluorescence signals of HA‐Cy5 adhesion on the rat femoral condyle or tibia platform on days 3 and 10. (g) Quantitative analysis of the fluorescence intensity of HA‐Cy5 and CS‐g‐PM+HA‐Cy5 group.

Transferring the construction strategy of HA coating in articular cartilage to the applications in vivo is necessary for therapeutic purposes. The retention time of HA in the joint cavity greatly affects its therapeutic efficacy. Therefore, to generate the composite coating of CS‐g‐PM+HA, we propose a sequential injection strategy, in which CS‐g‐PM is injected into the joint cavity first to obtain the CS‐g‐PM coating, and HA is then injected. We used in vivo imaging systems (IVISs) to evaluate the in vivo efficacy of HA binding and retention in a joint cavity environment (Figure 4a). It has been reported that cationic polyelectrolytes can be adsorbed on the surface of negatively charged rich articular cartilage, giving them a long retention time in the joint cavity [25]. We first examined the retention time of CS‐g‐PM labeled with Cy5 in the joint cavity of rats. As shown in Figure 4b,c, the fluorescence of CS‐g‐PM persisted for 6 weeks (42 days), indicating that CS‐g‐PM can maintain a consistent level of lubrication for an extended period. In addition, to demonstrate whether CS‐g‐PM adsorbed to the surface of articular cartilage, we dissected the rats on days 14 and 32 to obtain the femoral condyle and tibial plateau, respectively. Figure 4c,d found that compared with the PBS group, fluorescence signals could be detected in the cartilage of both the femoral slide and the tibial plateau in the CS‐g‐PM group, indicating that CS‐g‐PM could be successfully adsorbed onto the surface of the cartilage to form a lubricating coating of CS‐g‐PM, which is a prerequisite for the construction of the HA coating. Further, we examined whether CS‐g‐PM was beneficial in enhancing the retention of HA labeled with Cy5 in the joint cavity. The time‐course imaging of fluorescence revealed that the combination of CS‐g‐PM with sequential HA‐Cy5 injection remarkably prolonged the longevity of HA in the joint for up to 10 days (2 days in control HA‐Cy5 alone, Figure 4e), and the fluorescence intensity at each time point after injection was higher than that of HA alone (Figure 4g). Similarly, fluorescent signals of HA were detected on the surface of rat articular cartilage on days 3 and 10 in the CS‐g‐PM +HA group, but not in the HA group (Figure 4f), suggesting that CS‐g‐PM increased the adhesion of HA on the surface of articular cartilage, which in turn prolonged the retention time of HA in the joint cavity. The CS‐g‐PM bridge allowed the HA to be fixed to the rat knee's cartilage surfaces through electrostatic interactions. Consequently, the electrostatic self‐assembly approach enhanced HA retention in the knee's complex and hostile environment, extending the potential biological and physical advantages.

### 2.5 Radiographic Assessment of OA

**FIGURE 5:**
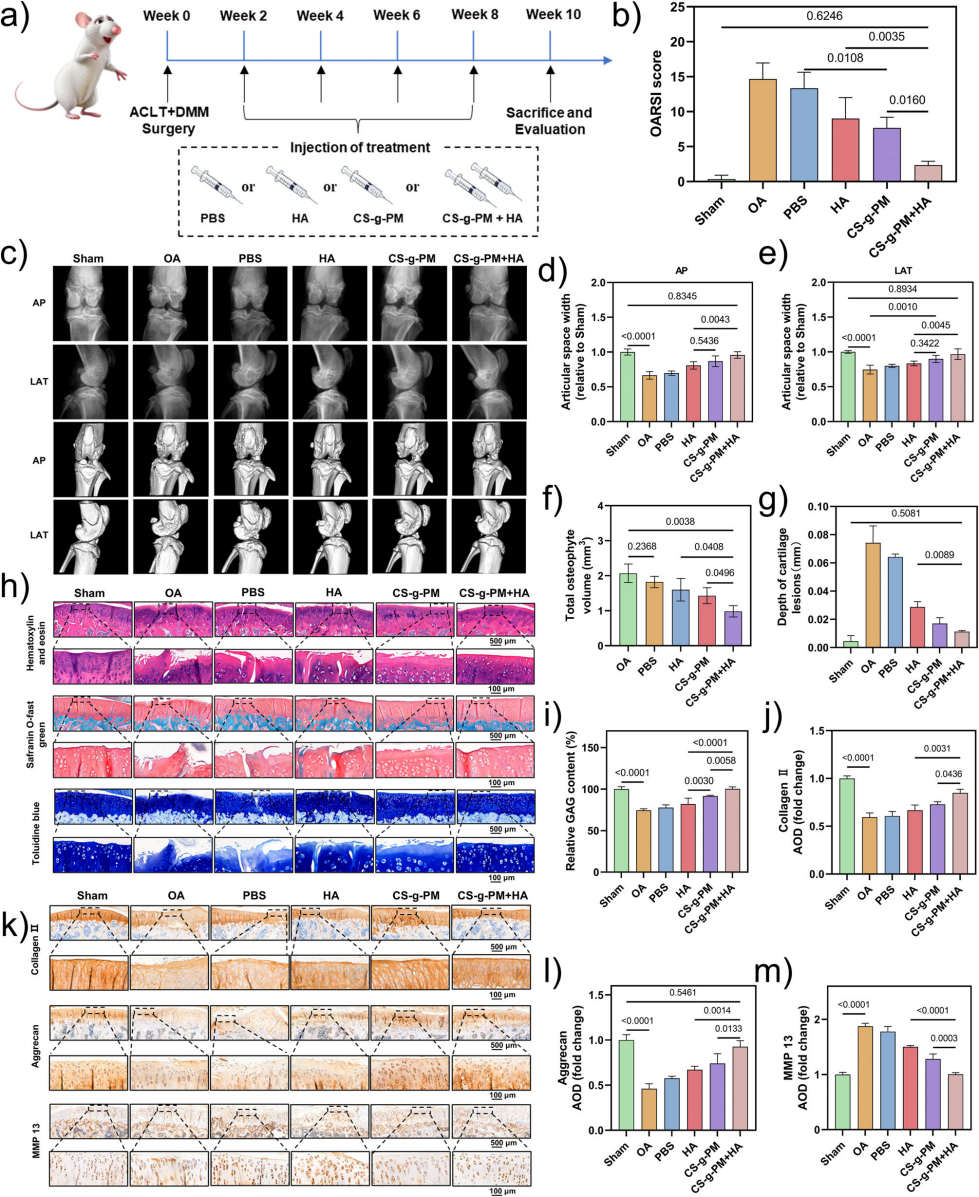
Evaluation of the chondroprotective effect and ability of HA, CS‐g‐PM, or CS‐g‐PM+HA to treat OA in rats. (a) Schematic illustration depicting the evaluation of the chondroprotective effect utilizing knee joints in rats with OA. (b) Cartilage OARSI score. (c) Representative X‐ray and Micro‐CT 3D reconstruction images after 8 weeks of joint cavity injection. (d) The quantitative analysis of JSWs from AP images. (e) The quantitative analysis of JSWs from LAT images. (f) Total osteophyte volume was serially outlined in sequential coronal sections and quantified from 3D reconstruction images. (g) Depth of cartilage erosion in H&E‐stained sections. (h) Representative histological stains, including H&E staining, Safranin O‐fast green staining, and Toluidine Blue staining, from each group. (i) Quantification of the relative content of glycosaminoglycans (GAG) in Safranin O‐fast green‐stained sections. (k) Representative immunohistochemistry staining of Col II, aggrecan, and MMP 13. (j, l, m) Quantitative analysis of relative Col II, aggrecan, and MMP13 protein expression. (*n* = 4, the values are shown as mean ± standard deviation (SD)).

The knee joints were surgically induced with OA over 2 weeks, followed by injection of PBS, HA, CS‐g‐PM, and CS‐g‐PM+HA to assess the therapeutic efficacy (Figure 5a). The rats were euthanized following OA‐inducing surgery in the eighth week. Subsequently, the knee joints underwent X‐ray radiography and Micro‐CT scanning. Because osteoarthritic cartilage loss typically manifests as a narrowing of the joint space, X‐ray images are frequently used to quantify joint space widths (JSWs) in order to track the development of the disease [38]. The results, in Figure 5c–e, demonstrated that the OA groups exhibited significantly narrower anterior‐posterior (AP) and lateral (LAT) joint space widths compared to the sham group, illustrating the successful establishment of the surgical models of OA in rats. Amongst the treatment groups, variations in JSWs were observed, with the narrowest width found in the PBS group, followed by HA and CS‐g‐PM groups. Notably, the CS‐g‐PM+HA group showed a significantly wider osteoarthritic joint space compared to the HA group, but no significant difference when compared to the sham group. The results suggest that the CS‐g‐PM+HA group can effectively slow down the progression of OA by enhancing the lubrication of articular cartilage and prolonging the retention time of HA. Osteophyte formation represents another pathogenic feature of OA and is considered to be a sort of compensation for joint injury [39]. Micro‐CT is a commonly used characterization of OA, as it can analyze the osteophyte formation through 2D imaging and 3D reconstruction. To evaluate therapeutic effects on OA more comprehensively, the changes in knee joint osteophyte burden were further analyzed using Micro‐CT imaging. In Figure 5c of 3D reconstructed images, the results revealed the absence of apparent osteophyte formation in the sham group, while present in the OA groups. It was evident that the CS‐g‐PM+HA group displayed improved joint morphology with reduced bone fragmentation. As illustrated in Figure 5f, compared with the OA group, the total osteophyte volume (TOV) of the CS‐g‐PM+HA group significantly decreased, followed by the CS‐g‐PM group, and no significant reduction in the PBS group. Importantly, the combined treatment of CS‐g‐PM and HA demonstrated a significant inhibition of osteoid formation compared to the individual treatments with HA or CS‐g‐PM alone, indicating a synergistic effect. These findings are consistent with the X‐ray images, providing evidence that CS‐g‐PM+HA is more efficacious in treating OA and mitigating its progression.

To assess cartilage structural changes, histological staining with hematoxylin and eosin, Safranin O‐fast green, and Toluidine blue was performed on rat knee joints after 8 weeks of joint cavity injection, followed by histopathological scoring across different groups. The primary pathological changes associated with OA include cartilage fissure, chondrocyte degeneration, and necrosis. The sham group, as depicted in Figure 5h, exhibits a smooth and continuous cartilage surface with a well‐defined structure, characterized by regularly and tightly arranged chondrocytes. In contrast, the articular cartilage of the OA and PBS groups appears to have typical features of OA, including rough and cracked cartilage, a disorganized structure, and evident cellular abnormalities. The other experimental groups exhibited improvement in morphological changes compared with the PBS group, with particular emphasis on the CS‐g‐PM+HA group, which demonstrated the best results in terms of cartilage integrity. Moreover, the HA group had greater cartilage wear than the CS‐g‐PM group, whereas the CS‐g‐PM+HA group had smooth cartilage surfaces and the least wear, and showed no significant difference with the sham groups. Compared with the OA group, the depths of the cartilage macroscopic lesion exhibited reductions of 61.32%, 77.04%, and 84.82% in the HA group, CS‐g‐PM group, and CS‐g‐PM+HA group, respectively (Figure 5g). Based on the color depth observed from Safranin O‐fast green staining, the CS‐g‐PM+HA group exhibited the highest amount of glycosaminoglycan, followed by the CS‐g‐PM group and HA group. This suggests that the CS‐g‐PM+HA group demonstrates superior potential in maintaining cartilage matrix integrity (Figure 5i). The results of OARSI scores are depicted in Figure 5b. In comparison to the PBS groups, HA groups, and CS‐g‐PM groups exhibited varying degrees of decreased OARSI scores, with the CS‐g‐PM+HA group demonstrating the most pronounced reduction, indicating a synergistic effect in mitigating cartilage damage. Immunohistochemical staining of Col II, aggrecan, and MMP 13 was also performed to investigate changes in cartilage‐associated matrix in the OA rats (Figure 5k). The expression of Col II and aggrecan showed improvement in rats treated with CS‐g‐PM alone or HA alone, with the CS‐g‐PM group exhibiting higher levels compared to the HA group. Compared to either CS‐g‐PM alone or HA alone, there was a greater density and continuity observed for Col II and aggrecan in the CS‐g‐PM+HA groups. Furthermore, in Figure 5j–l, quantitative analysis of Col II and aggrecan revealed that the CS‐g‐PM+HA group exhibited the highest expression, which was significantly different from that of the HA alone and CS‐g‐PM alone groups, further suggesting a synergistic effect of the combination of CS‐g‐PM and HA. In terms of the expression of inflammatory factor MMP 13, there was no significant difference observed between the CS‐g‐PM+HA group and the sham group, but both were lower levels compared to the HA or CS‐g‐PM groups (Figure 5m). Overall, Enrichment of HA on the cartilage surface via electrostatic self‐assembly can effectively increase the lubricating properties of cartilage and prolong the retention time of HA, reducing joint wear and slowing the progression of OA, providing a new viable strategy to increase the efficacy of HA in clinical practice.

### 2.6 First‐in‐Human Pilot Observation of Proposed Stepwise Injection Strategy

The three case reports presented below are provided solely as preliminary, descriptive examples from a single‐blind study, intended to illustrate safety, feasibility, and potential efficacy. Their presentation does not involve unblinding of the full trial dataset, nor does it constitute any formal interim analysis of efficacy between treatment groups.

Case 1, a 60‐year‐old female, presented with left knee pain and functional limitation for over 5 years. The patient had received nonsteroidal anti‐inflammatory drug (NSAID) treatment but failed to achieve adequate symptomatic control, especially while going down stairs. According to the inclusion criteria of the randomized controlled trial, the patient was allocated to the hyaluronic acid (HA) treatment group. The Western Ontario and McMaster Universities Osteoarthritis Index (WOMAC)—a validated, widely used tool for quantifying osteoarthritis (OA)‐related pain, stiffness, and functional impairment—was serially assessed for Case 1, Case 2, and Case 3 at baseline, 1 and 6‐month follow‐ups, with detailed scores summarized in Table 1. At baseline, case 1 presented with a total WOMAC score of 26. At 1‐month follow‐up after HA injection, significant symptomatic amelioration was observed, with the total WOMAC score decreasing to 8, indicating that pain and joint function have shown significant improvement in the short term. However, at the 6‐month follow‐up, the total score increased to 24, indicating a recurrence of symptoms, particularly in stiffness and functional domains.

**TABLE 1:** The WOMAC score, CRP, ESR, and IL‐6 of the three cases.

		WOMAC				CRP	ESR	IL‐6
		Pain	Stiffness	Function	Total			
Case 1	Before	9	0	17	26	20.1	19	29.55
	1‐month	2	0	6	8	3.01	20	1.5
	6‐month	2	6	16	24	4.5	22	< 1.5
Case 2	Before	12	20	73	105	27.79	28	16.28
	1‐month	2	10	38	50	< 3	10	5.669
	6‐month	1	6	22	29	< 3	11	< 1.5
Case 3	Before	16	8	58	82	< 3	9	< 1.5
	1‐month	3	2	19	24	< 3	10	< 1.5
	6‐month	1	0	10	11	< 3	7	< 1.5

Magnetic resonance imaging (MRI) assessment (Figure S8) similarly corroborated these clinical findings, revealing no discernible signs of cartilage repair at both the 1 and 6‐month follow‐ups—consistently aligning with the clinical outcomes reflected in WOMAC scores. This outcome aligns with the typical characteristics of hyaluronic acid injection therapy alone: effective short‐term symptom relief but lacking sustained structural improvement.

**FIGURE 6:**
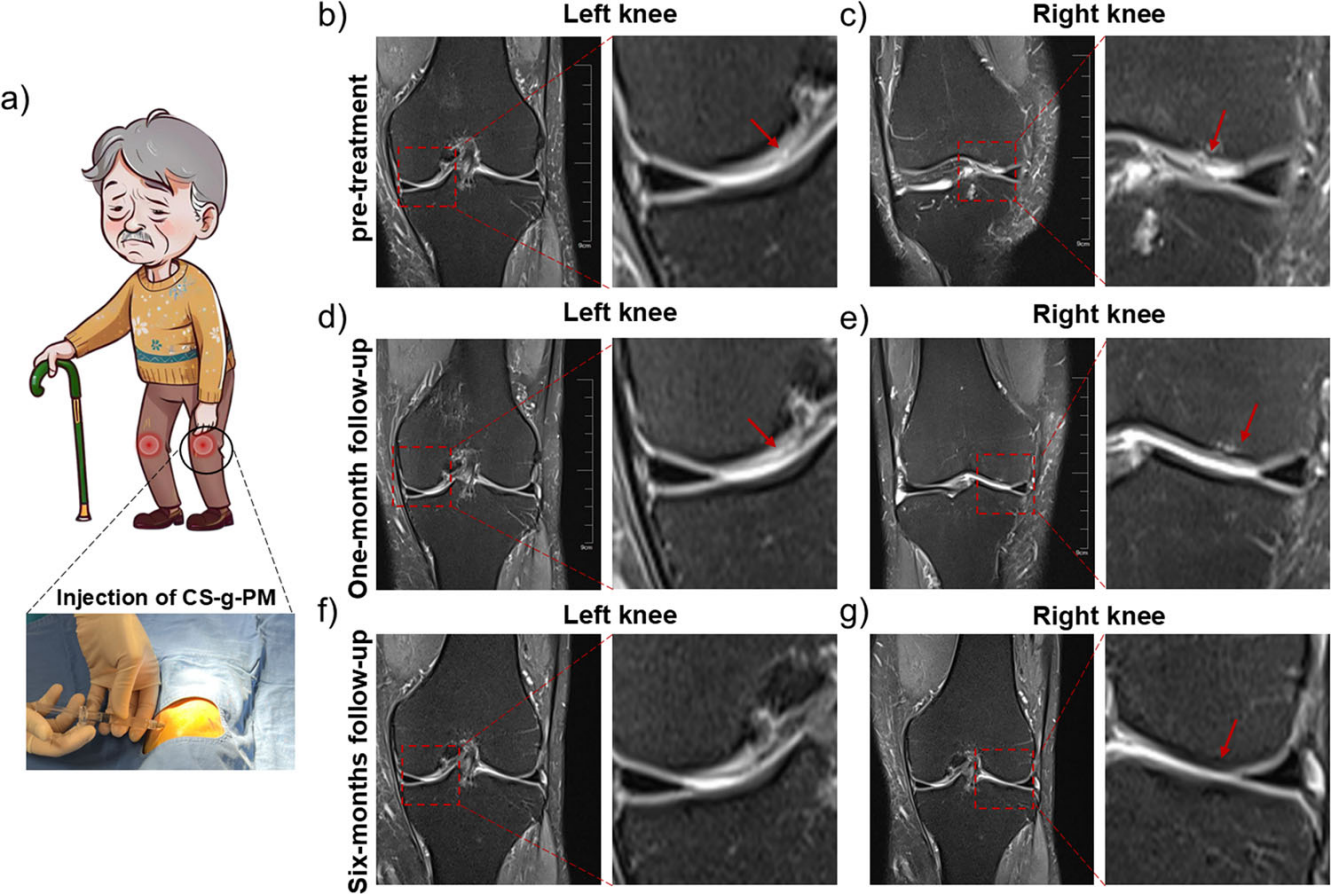
Comparison of MRI for pre‐ and post‐treatment for Case 2 in clinical trials. (a) Patient with OA in both knees and a schematic diagram of CS‐g‐PM intra‐articular injection. The coronal standard MRI of bilateral knee, before (b,c), 1‐month follow‐up (d,e), and 6‐month follow‐up (f,g) after the injection. Red arrows show significant improvement in the cartilage of the knee joint.

A 78‐year‐old male patient with no history of knee trauma or surgery met the inclusion criteria and was enrolled in the clinical trial. His medical history included chronic oral nonsteroidal anti‐inflammatory drug (NSAID) and glucosamine sulfate use, with no prior intra‐articular hyaluronic acid (HA) injections. Based on the randomization protocol, the patient was allocated to the CS‐g‐PM treatment group. The patient experienced improvement in pain relief after the third injection in both knees. In Table 1, the WOMAC score was rated 105 at the beginning of injection, which was down to 50 after 1 month of follow‐up. Surprisingly, at the 6‐month follow‐up, the patient's WOMAC score was further reduced to 29. Especially, in the blood test of C‐reactive protein (CRP), erythrocyte sedimentation rate (ESR), and interleukin‐6 (IL‐6), the inflammatory factor significantly decreases to a normal level over the course of follow‐up. The MRI of the patient's bilateral knee joints was performed at baseline, 1 and 6 months post‐initiation of therapy in Figure 6b–g. Pre‐intervention MRI revealed areas of high signal intensity within the articular cartilage (red arrows), indicative of cartilage injury. Post‐treatment MRI examinations (at 1 and 6 months) demonstrated imaging features suggestive of cartilage repair at the injury sites. Specifically, the 6‐month follow‐up MRI showed a reduction in the intensity of focal signal abnormalities within the articular cartilage and subchondral bone marrow, approaching the signal characteristics of adjacent normal cartilage. Compared to the pre‐treatment and 1‐month MRI, no progression of cartilage damage was evident on the 6‐month images, suggesting stabilization of cartilage wear.

**FIGURE 7:**
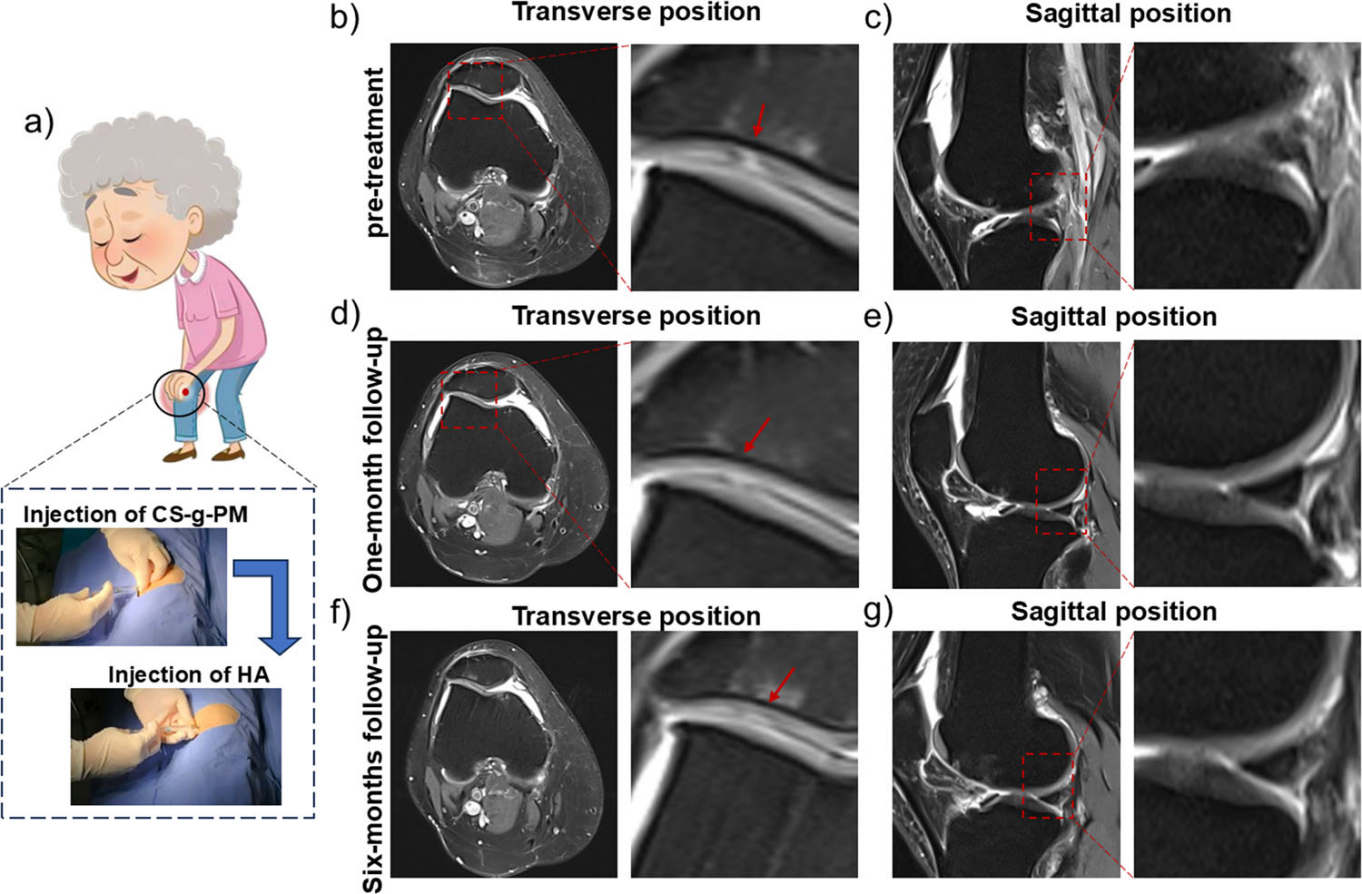
Comparison of MRI for pre‐ and post‐treatment for Case 3. (a) Patient with OA in the right knee and schematic diagram of CS‐g‐PM+HA intra‐articular injection. The coronal standard MRI of the right knee, before (b,c), 1‐month follow‐up (d,e), and 6‐month follow‐up (f,g) after the injection. Red arrows show significant improvement in the cartilage of the knee joint.

A 63‐year‐old female, case 3, suffered from right knee pain for over 4 years, had a mildly limited range of motion in her knee, but aggravated pain while going down stairs, and she had not received any medication or physical therapy. The patient was randomized to the CS‐g‐PM+HA group based on the inclusion criteria of the randomized controlled study. As shown in Table 1, the patient's WOMAC score was 82 before the injection, indicating moderate OA. Especially during the second injection, she reported relief of pain and decreased tenderness. After the fifth injection of the treatment, the patient reported the disappearance of knee joint pain when going down stairs, and the follow‐up data presented in Table 1 primarily demonstrate improvements in pain and function postoperatively, as indicated by a reduction in the WOMAC score from 82 to 24. At a longer 6‐month follow‐up, the patient's pain, stiffness, and functional scores improved significantly, and the WOMAC total score decreased to 11. However, no significant differences were observed in the levels of CRP, ESR, and IL‐6 in the blood tests. In Figure 7b–g, the sagittal and axial postoperative MRI scans revealed evidence of cartilage healing at the articulation of the medial femoral condyle and patella. MRI results at the 6‐month follow‐up showed that articular cartilage thickness, signal intensity, and articular surface integrity remained stable without significant degenerative changes compared with the 1‐month follow‐up.

Notably, the clinical efficacy demonstrated for both CS‐g‐PM and CS‐g‐PM+HA in these cases reveals significant advantages over established intra‐articular HA therapy. Consistent with the literature [40–42], HA injections typically yield a more modest improvement in WOMAC scores, often cited in the range of 30%‐50% within 3–6 months, with peak effects frequently observed at 1–3 months followed by attenuation thereafter. In contrast, CS‐g‐PM therapy achieved a sustained 72.4% reduction in WOMAC score at 6 months (105–29), while the CS‐g‐PM+HA therapy exhibited an even more profound 86.6% reduction (82–11). In terms of cartilage repair, both CS‐g‐PM and CS‐g‐PM+HA groups displayed clear imaging evidence of cartilage repair with the 1 and 6‐month MRI. These findings suggest that CS‐g‐PM and CS‐g‐PM+HA not only provide superior and longer‐lasting pain relief and functional improvement compared to HA therapy but also offer the unique advantage of promoting cartilage repair, addressing both symptoms and underlying structural damage in osteoarthritis.

The clinical outcomes observed in these representative cases support the feasibility, safety, and potential therapeutic efficacy of intra‐articular CS‐g‐PM and CS‐g‐PM + HA injections for knee osteoarthritis. This ongoing clinical trial will provide a comprehensive statistical analysis of efficacy and safety outcomes from the multicenter cohort in forthcoming reports.

## 3 Discussion

In this study, we have developed a strategy for constructing interface‐mimetic synergistic lubrication coatings, which significantly enhance the therapeutic outcome of HA for OA treatment. Inspired by the natural cartilage lubrication interface, we designed a cationic biomimetic lubricant, CS‑g‑PM, by grafting phosphorylcholine‐rich polymer brushes onto a chitosan backbone. CS‑g‑PM not only mimics the cartilage‐anchoring function of lubricin but also incorporates hydration lubrication capability via phosphorylcholine side chains. Through a stepwise intra‑articular injection protocol, CS‑g‑PM first adsorbs electrostatically onto the negatively charged cartilage surface, subsequently capturing free HA to form a stable, surface‐bound lubrication coating via in situ self‐assembly. This coating recapitulates the structural and functional features of the natural lubricating complex, thereby overcoming the key limitations of conventional HA therapy. We demonstrated that the CS‑g‑PM+HA coating synergistically restored the lubrication properties of OA human cartilage in vitro, reducing the friction coefficient to near‐physiological levels. The enhanced performance is attributed to the combined action of boundary lubrication provided by surface‐enriched HA and hydration lubrication rendered by the phosphorylcholine brushes of CS‑g‑PM. In vivo, CS‑g‑PM acted as a molecular bridge that anchored HA to the cartilage surface, extending its articular retention fivefold compared to HA alone. In a rat OA model, sequential administration of CS‑g‑PM and HA exerted a synergistic chondroprotective effect, significantly inhibiting osteophyte formation, reducing cartilage erosion, and enhancing the synthesis of cartilage‑specific extracellular matrix components, including type II collagen and aggrecan.

Importantly, the clinical relevance of this strategy was preliminarily validated in a first‑in‑human pilot trial. Patients receiving the CS‑g‑PM‐based stepwise injection exhibited rapid improvement in joint function and pain relief, with MRI evidence of cartilage repair observed as early as 1 month post‑treatment. At 6‑month follow‑up, both symptomatic relief and structural stabilization were sustained, indicating that the biomimetic lubrication coating not only prolongs HA residence but also actively promotes a reparative joint microenvironment. These outcomes contrast sharply with the transient symptomatic benefit and lack of structural improvement typically associated with standard HA injections. The comprehensive statistical analysis of the full cohort (including WOMAC scores and MRI quantification) will be reported in a separate, dedicated clinical publication upon the completion and unblinding of the RCT. Collectively, this work moves beyond mere lubrication replacement toward active cartilage surface regeneration. By leveraging electrostatic self‐assembly to construct a durable, biomimetic lubrication interface, our strategy addresses the challenges of poor HA retention. In addition, Engineered HA‐based microcarriers have emerged as an alternative strategy for OA treatment, offering advanced platforms for cell and drug delivery [43]. The future work could combine these two strategies, where our strategy goes beyond standalone lubrication therapy to synergistic regenerative treatment. The CS‑g‑PM priming injection represents a simple yet powerful modality to transform conventional HA therapy into a regenerative treatment, offering a clinically feasible and scalable approach to halt OA progression. Future large‑scale randomized controlled trials will further quantify the long‑term efficacy and safety of this interface‑bionic strategy, with the potential to establish a new paradigm in OA management.

## 4 Materials and Methods

### 4.1 Preparation of Lubricant Coatings on Cartilage

Articular cartilage lubricating coatings were prepared by electrostatic self‐assembly. The fresh pig knee joints were obtained, and the cartilage was harvested from the femoral condyles using a 7 mm diameter cyclic drill. The cartilage explants were rinsed with sterilized PBS and promptly utilized for experimentation. Due to the existence of abundant negatively charged, cationic polyelectrolytes, they were easily adsorbed on the cartilage surface. First, the articular cartilage was immersed in 1 mg/mL CS‐g‐PM solution for 1h, and after washing with PBS, the articular cartilage with CS‐g‐PM coating was obtained. Then, it was immersed in 1 mg/mL HA solution for 1h so that HA was adsorbed on the cartilage surface, and after rinsing with PBS, the composite coating with CS‐g‐PM and HA was obtained.

### 4.2 Characterization of Lubricant Coatings

The microscopic morphology and roughness of the coatings were observed by SEM (Merlin, Zeiss, Germany) and 3D profilometer (Sneox, Sensofar, Spain) after lyophilizing cartilage samples. FTIR (iS50, Thermo, German) and XPS (Escalab, Thermo, German) were performed to verify the presence of the lubricant coating on articular cartilage; The hydrophilicity of the lubricating coating was tested by a drop shape analyzer (DSA25E, Kruss, German).

### 4.3 Visualization of Lubricant Coatings

The HA and CS‐g‐PM samples were labeled with 6‐AF and rhodamine B, respectively. Briefly, 0.1 g of HA was dissolved in 100 mL of distilled water, followed by the sequential addition of EDC (15 mg) and NHS (10 mg). After a 30‐min incubation period, 5 mg of 6‐AF was added to the HA solution and stirred for 24 h in the dark. Subsequently, the mixture was centrifuged at 5000 rpm for 10 min, and the resulting supernatant was dialyzed in distilled water under dark conditions for a duration of 3 days. Finally, HA‐6‐AF conjugates were obtained through lyophilization. Rhodamine B labelled CS‐g‐PM was obtained in the same way (CS‐g‐PM‐RhB).

The articular cartilage lubricating coating was prepared according to the above steps. According to our previous method [6], all cartilage samples were observed by confocal laser scanning microscopy (TCS SP8, Leica, Germany).

### 4.4 Friction Measurements on Human Cartilage Samples

The studies were conducted in accordance with the guidelines set forth by the Institutional Review Board of Guangdong Hospital of Traditional Chinese Medicine (ZE2023‐418‐01). Normal human articular cartilage samples were isolated from the patients with ages 54 (M), 77 (F), 58 (M), 80 (F), and 60 (F), who underwent total knee arthroplasty or hip replacement surgery. For the isolation of human cartilage samples, a hollow drill was used. The drill was perpendicular to the surface of the cartilage. The samples were vigorously washed in PBS. Then, the samples were subjected to treatment with 0.5% trypsin solution for 3 h to mimic OA as previously reported [44]. The articular cartilage lubricating coating was prepared on the mimic OA cartilage, and lubrication testing in PBS was performed. The tribological test used the universal mechanical tester (UMT TriboLabTM, Bruker, Germany) [45]. The top (5 mm, diameter) and bottom (7 mm, diameter) friction pair consisted of circular human cartilage samples with or without coatings. The lubrication of coatings was assessed by measuring the coefficient of friction (COF) under the following test conditions: shear rates ranging from 0.5 to 5.0 mm/s and a load range of 1–3 N, with 5 repetitions of each set averaged. Furthermore, we tested the friction coefficient of cartilage under different loading times. In short, the friction coefficient of cartilage was measured after it was subjected to a fixed load of 1 N for different periods of time (120, 12, and 1.2 s) before sliding. Each group was measured five times.

### 4.5 Safety Assessment of Intra‐Articular Injections

Permission was granted to perform animal studies by the South China University of Technology Animal Care and Use Committee (2023050). To evaluate the safety of intra‐articular injection of CS‐g‐PM and CS‐g‐PM+HA, 15 normal New Zealand rabbits were selected and randomly divided into 5 groups of 3 rabbits each, which were divided into Normal, PBS, HA, CS‐g‐PM, and CS‐g‐PM+HA groups according to the different materials injected into the intra‐articular cavities, and the remaining five groups, except for the Normal group. The rabbits were injected with 300 µL of the corresponding materials in the right knee, and then tissue sampling was performed after 1 month of rearing. After the experimental animals were kept for 1‐week, appropriate amounts of blood were collected by vacuum anticoagulation tubes for routine blood tests. Liver, kidney, and right knee joint tissues were collected, labelled, and fixed in 4% paraformaldehyde.

### 4.6 In Vivo Imaging for Retention Time

Permission was granted to perform animal studies by the South China University of Technology Animal Care and Use Committee (2023050). First, Cy5‐tagged CS‐g‐PM and HA were prepared by the reaction of amino groups with NHS esters. CS‐g‐PM (50 mg) was dissolved in PBS, and after adjusting the pH = 8 with NaOH, Cy5‐NHS (1 mg) was added and reacted for 2 h in the dark. Then, the mixture was precipitated dropwise into excess ethanol, and CS‐g‐PM‐Cy5 was obtained by centrifugation, washing, and lyophilization. HA was modified with an amino group (HA‐ADH) according to a previous report [6], then dissolved in PBS to prepare a 1 mg/mL solution. Cy5‐NHS was put into the HA solution to form HA‐Cy5.

In vivo imaging was conducted on 4–6‐week‐old male Sprague Dawley rats (*n* = 5 for each group). The rats were anesthetized with isoflurane under a pre‐established protocol (The South China University of Technology Animal Care and Use Committee approved the animal procedures, protocol number 2023050). The rats in the OA group were anesthetized, followed by anterior cruciate ligament transection and medial meniscus resection to induce OA. Two weeks post‐surgery, the OA rats were further randomized into four groups: 100 µL PBS, 100 µL CS‐g‐PM‐Cy5, 50 µL PBS+50 µL HA‐Cy5(sequential injection, same below), and 50 µL CS‐g‐PM+50 µL HA‐Cy5. After joint cavity injection, rats were subjected to imaging and kept under isoflurane anesthesia utilizing an IVIS Spectrum in vivo imaging system (IVIS Spectrum, PerkinElmer, America) at different time points. All images were captured using identical excitation (640 nm) and emission (680 nm) settings. Prior to each imaging session, the rats underwent anesthesia and hair removal.

### 4.7 Rat Model of OA

Permission was granted to perform animal studies by the South China University of Technology Animal Care and Use Committee. Male Sprague Dawley (SD) rats, aged 8 weeks, were randomly allocated into two groups: the sham group (*n* = 4) and the OA group (*n* = 20). The rats in the OA group were anesthetized, followed by anterior cruciate ligament transection and medial meniscus resection to induce OA. Two weeks post‐surgery, the OA rats were further randomized into five groups: OA model group without further treatment; 100 µL PBS group; 100 µL HA(1 mg/mL) group; 100 µL CS‐g‐PM(1 mg/mL) group; and 50 µL CS‐g‐PM(1 mg/mL)+50 µL HA (1 mg/mL) group. The injections were performed once every 2 weeks for a total duration of 8 weeks.

### 4.8 Clinical Trial Design and Intervention

This study is approved by the Chinese Clinical Trial Registry (ChiCTR) with the code ChiCTR2400086434. The study was a single‐blind, randomized, and controlled study designed to assess the long‐term effects of a 5‐week course of weekly intra‐articular injection. All patients were recruited from the hospital outpatient department and provided written informed consent for data collection and review. Patients were randomly divided into three groups: the HA group, treated with 2.5mL Hyprojoint (Sodium Hyaluronate, 2.5 mL:25 mg, Bloomage Biotech, China); the CS‐g‐PM group, injected with 2.5 mL Biomimetic Lubricants (CS‐g‐PM, 2.5 mL:2.5 mg, Guangzhou Proud Seeing Biotechnology Co., Ltd., China); and the CS‐g‐PM+HA group, which received sequential injection of 1.25 mL CS‐g‐PM + 1.25 mL Sodium Hyaluronate. The CS‐g‐PM used was manufactured under GMP conditions by Guangzhou Proud Seeing Biotechnology Co., Ltd., according to our proprietary synthesis protocol. In this single‐blind design, the patients, as well as the MRI assessors and data analysts, were blinded to treatment allocation throughout the study period. The treating physicians were necessarily aware of the assigned intervention at the time of administration due to the procedural differences among groups—particularly the two‐step sequential injection required in the CS‐g‐PM+HA group, as opposed to the single‐injection regimens used in the other groups. Each patient underwent a standardized procedure of intra‐articular injection via the suprapatellar lateral approach, administered weekly for a total of five sessions. Concurrently, patients were given 200 mg of Celecoxib (CSPC Pharmaceutical Group Limited, China) and 10 mg of Omeprazole (Hainan Hailing Chemipharma Corporation Limited, China), both once daily, for the treatment of knee OA.

### 4.9 Ethical Considerations

The study of intra‐articular injection of CS‐g‐PM was approved by the Medical Ethics Committee of Zhongshan Hospital of Traditional Chinese Medicine, with the approval number of 2024ZSZY‐LL‐KY‐005 (Chinese Clinical Trials Registry, registered on 1 July 2024). Participants in this clinical trial will be covered by clinical trial insurance, which will offer suitable protection if they suffer serious harm.

To preliminarily evaluate the safety of intra‐articular injection of CS‐g‐PM, we conducted an early clinical observation on the first three patients enrolled in the ongoing RCT. This clinical observation was approved by the Medical Ethics Committee, and the patients were informed that the available clinical data would be used solely for case reports without affecting the progress of the RCT.

### 4.10 Assessment

The initial analysis was conducted prior to patient enrollment, and the follow‐up analysis was performed at 1 and 6‐month post‐injection. A functional evaluation, the Western Ontario and McMaster Universities OA (WOMAC) index, was used to assess the patients’ symptoms and functional impairments of knee OA. The index, which ranges from 0 to 96, is divided into three primary sections: pain (20 points), stiffness (8 points), and functional disability (68 points). Its validity and reliability have been extensively confirmed through multiple validation studies. Additionally, inflammatory markers, including CRP, ESR, and IL‐6, were measured for evaluation at the same time. The whole organ magnetic resonance imaging was used for radiological assessment, of which five features related to articular surfaces were examined, including cartilage signal and morphology, subarticular bone marrow abnormalities, subarticular bone cysts, subarticular bone attrition, and marginal osteophytes.

### 4.11 Statistical Analysis

The data obtained were represented as the mean ± standard deviation. Statistical analysis was performed using analysis of variance to determine significance levels (^*^ *p* < 0.05, ^**^ *p* < 0.01, ^***^ *p* < 0.001, ^****^ *p* < 0.0001), while non‐significant results were denoted as NS.

## Author Contributions

Y.L., R.X., M.D., Z.C., and L.R. supervised the project. Y.L. and Z.Y. carried out the animal experiments and provided fruitful discussion on the results of the animal experiments. J.C. carried out a clinical trial with the assistance of Z.Y., Z.L., and B.W. X.W. carried out clinical sampling of cartilage from patients with OA under the supervision of X.C. Y.L., R.L., and Y.F. carried out the rest of the experiments and characterization. All the authors contributed to the discussions and writing of the manuscript.

## Funding

This work was supported by the National Natural Science Foundation of China (52173123, 52303180), the Guangdong Province Key Field R&D Program Projects (2020B1111150002, 202206010160), the Guangdong Basic and Applied Basic Research Fund (2022B1515230008), Major Project of Guangzhou National Laboratory (GZNL2024A03006), Jiangxi Provincial Natural Science Foundation (20252BAC220026) and Jiangxi Province Thousand Talents Project (jxsq2023101065).

## Conflicts of Interest

The authors declare no conflicts of interest.

## Supporting information




**Supporting File**: advs75195‐sup‐0001‐SuppMat.docx.

## Data Availability

The data that support the findings of this study are available from the corresponding author upon reasonable request.
