# Supplementary material for: A Biomimetic Lubricant Captures Hyaluronic Acid In Situ to Regenerate Cartilage: From Bench to Bedside
Source: Adv Sci (Weinh). 2026 Apr 10:e00049. Online ahead of print. doi: 10.1002/advs.202600049 (PMC13334645; doi:10.1002/advs.202600049)
Supplement: Supplementary file 1 — Supporting File: advs75195‐sup‐0001‐SuppMat.docx. [file ADVS-9999-e00049-s001.docx]

**Supporting Information**

**A Biomimetic Lubricant Captures Hyaluronic Acid In Situ to Regenerate Cartilage: From Bench to Bedside**

Yongan Lin^1, 2, 6, 8, †^, Zijian Yan^1, 2, †^, Jiayi Chen^3, †^, Xiaochao Wang^4^, Ruibin Lin^1, 2^, Yunqi Fu^1, 2^, Zhaoying Lv^3^, Bohui Wu^3^, Xuewei Cao^4^, Renjian Xie^7, *^, Ming Dong^5, 6, *^, Chenxiao Zheng^3, *^, Li Ren^1, 2, *^

1 School of Materials Science and Engineering, South China University of Technology; Guangzhou 510006, China

2 National Engineering Research Center for Tissue Restoration and Reconstruction; Guangzhou 510006, China

3 Zhongshan Hospital of Traditional Chinese Medicine Affiliated to Guangzhou University of Traditional Chinese Medicine; Zhongshan 528400, China

4 Orthopaedic Hospital of Guangdong Provincial Hospital of Traditional Chinese Medicine; Guangzhou 510006, China

5 State Key Laboratory of Respiratory Disease, the First Affiliated Hospital of Guangzhou Medical University, Guangzhou Medical University; Guangzhou, 510120, China

6 Guangzhou National Laboratory, No. 9 XingDaoHuanBei Road, Guangzhou International BioIsland; Guangzhou 510005, China

7 School of Medical Information Engineering, Gannan Medical University, Ganzhou 341000, China

8 Guangzhou Proud Seeing Biotechnology Co., Ltd., Guangzhou 510623, China

† These authors contributed equally to this work

* Renjian Xie, Ming Dong, Chenxiao Zheng, Li Ren, E-mail address: psliren@scut.edu.cn.

Experimental Section

Materials

Sodium hyaluronate (HA, Mw: 1.0–1.5×10^6^ Da) was obtained from Shanghai Yuanye Biological Technology. N-hydroxy succinimide (NHS), N-ethyl-N-(3-(dimethylamino) propyl) carbodiimide (EDC), glycidyl methacrylate (GMA), 6-Aminofluorescein (6-AF, ≥97%), Rhodamine B (RhB) and potassium persulfate were purchased from Aladdin (Shanghai, China). 2,2-methacryloyloxyethyl phosphorylcholine (MPC) was obtained from Jenkem Technology. Medical chitosan was purchased from ZHEJIANG GOLDEN-SHELL PHARMACEUTICAL CO., LTD. Cy5-NHS was purchased from Xi’an Ruixi Biological Technology.

Synthesis of cationic biomimetic lubricants (CS-g-PM)

Synthesis of double bond modified chitosan (CSMA): Medical chitosan (1 g) was dissolved in 250 mL of deionized water by adjusting the pH to 4 with hydrochloric acid. The solution was heated to 60°C, and glycidyl methacrylate (700 μL) was added dropwise. The reaction was allowed to proceed for 18 h. After reaction, the product was dialyzed against deionized water using a dialysis tubing with a molecular weight cutoff (MWCO) of 8,000–14,000 for 3 days. The resulting solution was freeze-dried to obtain double bond modified chitosan (CSMA).

Briefly, CSMA (0.2 g) was dissolved in deionized water (80 mL) and stirred until completely dissolved under 60 ℃. After the solution was purged with nitrogen for 1 h. Potassium persulfate (50 mg) and MPC (1 g) was dissolved in deionized water 6 mL and 20 mL. Then, the initiators and monomers are added dropwise to the above solution by peristaltic pump within 2h. At the end of the drop, the mixture was stirred for 4 h under N_2_ atmosphere. After that, the mixture was rapidly cooled to room temperature to stop the reaction. The solution was transferred to the dialysis tube with the 8 kDa-14 kDa molecular weight cut-off and dialyzed against deionized water for 3 d and then lyophilized to obtain CS-g-PM.

Chemical structure characterization of CS-g-PM

The products were investigated using nuclear magnetic resonance spectroscopy (NMR) and Fourier transform infrared spectrophotometry (FTIR). ^1^H NMR spectra were recorded on an NMR spectrometer (400 MHz, Bruker, Germany), with D_2_O as a solvent. The FTIR spectra of the CS, CSMA and CS-g-PM were produced by a Bruker Vector 33 FTIR spectrometer at room temperature in arrange of 4000 cm^-1^~500 cm^-1^ by the method of KBr tableting. A certain mass of CS-g-PM was weighed to prepare a solution of 1 mg/mL in deionized water, and then adjusted with hydrochloric acid or sodium hydroxide to pH=3, 4, 6 and 7. The zeta potential of CS-g-PM at different pH was tested by a zeta potential meter (Zetasizer Nano ZSE, Malvern, United Kingdom).

Characterization of the interaction between CS-g-PM and HA

To investigate the interaction between HA and CS-g-PM, quartz crystal microbalance with dissipation (E4, Q-sense, Sweden) was used. Firstly, clean gold chips were loaded into the QCM sample tank, passed through PBS, and after the baseline was run flat, 1.0 mg/mL of CS-g-PM was passed, and after adsorption was saturated, passed through PBS and rinsed, followed by the passage of 1.0 mg/mL of HA, rinsed with PBS, and then passed through 1.0 mg/mL of CS-g-PM again. The interaction between CS-g-PM and HA was reflected by the detection of the change of the frequency (Δf). Further, the interaction of HA with CS-g-PM was investigated by the change of zeta potential. Specifically, a 1 mg/mL solution of HA was prepared with deionized water. Complexes with different mass ratios of HA to CS-g-PM were obtained by dropwise addition of 0.1, 0.2, 1, 5, and 10 mg/mL of CS-g-PM at room temperature under stirring condition. The zeta potential of CS-g-PM and HA complexes was tested by a zeta potential meter (Zetasizer Nano ZSE, Malvern, United Kingdom).

Cytotoxicity and proliferation of chondrocyte cells

The C-28/I2 cell line, derived from immortalized human chondrocytes, was procured from Sigma-Aldrich and employed consistently in this investigation. C-28/I2 was cultured in a complete medium containing DMEM, FBS (10%), penicillin (100 UI/mL), and streptomycin (100 µg/mL). The cytotoxicity and proliferation of C-28/I2 cells were assessed using the Cell Counting Kit-8 (CCK-8, Dojindo, Japan) and the Live/Dead cell viability assay kit (Solarbio, Beijing, China) at various time intervals. In the CCK-8 assay, a solution of CCK-8(10 µL) was added to the culture medium and incubated for 4 hours. The absorbance was then measured at 450 nm using a microplate reader (Thermo Fisher Scientific, MA, USA). For the Live/Dead staining assay, calcein-AM/propidium iodide was applied to the cells for 20 minutes followed by examination under a fluorescence microscope (Axio Observer, Zeiss, German).

Properties of Antibacterial

Firstly, 2.1 g of LB medium was mixed with 100 mL of distilled water and then sterilized in a sterilization pot at 120 °C for 15 minutes to prepare a liquid medium. Similarly, 2.1 g of LB medium and 1.5 g of agar powder were mixed with 100 mL of distilled water and sterilized in a sterilizer at 120 ℃ for 15 minutes. When the medium was cooled down to 50 ℃, 15 mL of medium was pipetted onto a sterile disposable plate to prepare a solid medium. Add 3 mL of LB liquid medium into the bacterial culture tube, add the single colony screened out from the solid medium of E. coli or S. aureus into the liquid medium, and incubate for 24 hours in a thermostatic oscillator (37 ℃, 200 rpm), and then prepare the bacterial suspension. E. coli or S. aureus was diluted to 10^6^ CFU/mL in LB liquid medium, and then different concentrations of CS-g-PM were added to 200 μL of bacterial suspension, and the mixture was incubated at 37 ℃ for 6 hours. Finally, 100 μL of the diluted solution was evenly spread on an LB solid medium and placed in a 37 ℃ constant temperature incubator for 18 h. The medium was photographed and the colonies were counted, and the bacterial survival rate was calculated according to the following formula:

SR%=(BC in experiment group / BC average in control group)*100%

Radiographic evaluation

The rats were administered euthanized in the 8th week post-surgery. The isolated knee joint specimens were immersed in a fixative solution containing 4% paraformaldehyde for a duration of 24 hours. Subsequently, X-ray radiography was performed on the rat knee joints, and analysis of the knee space width across different groups of rats was conducted based on the obtained scan images. Next, Micro-CT scanning (Quantum GX2, PerkinElmer, America) was employed using a scanning voltage of 90 kV and a scanning current of 88 µA to reconstruct both subchondral bone and knee joints for osteophyte analysis

Histologic and immunohistochemical examinations

The knees were fixed with paraformaldehyde, decalcified, embedded in paraffin, and then sliced into 5 μm thick sections. Histological analysis was conducted by staining the sections with H&E staining, Safranin O-fast green staining and Toluidine Blue. An Osteoarthritis Research Society International (OARSI) score, which serves as an indicator of cartilage damage, is determined by an impartial scorer and based on a representative image captured from the medial plateau of the tibia. For immunohistochemical staining, Triton X-100/10% horse serum (0.2%, in PBS) was utilized to facilitate permeabilization and blocking of the joint samples. The sections were then subjected to overnight incubation at 4 ℃ with primary antibodies targeting Col Ⅱ/aggrecan (arigobio, China), followed by a subsequent 1-hour incubation with secondary antibodies. To determine the relative expressions of Col Ⅱ and aggrecan, ImageJ software was employed for quantification.

Inclusion and exclusion criteria

The inclusion criteria are as follow: 1) Patients who were diagnosed with knee osteoarthritis accompanied by cartilage defects through MRI examination. 2) No other autoimmune or systemic diseases. 3) Agree not to participate in other related studies, and do not accept other osteoarthritis treatment plans during the participation in this study. 4) No use of other drugs that may affect the effect of osteoarthritis during the treatment period. 5) Signed an informed consent form and can cooperate with the treatment plan of this project.

The exclusion criteria are as follow: 1) Suffering from severe systemic diseases other than osteoarthritis. 2) Having clinically significant urinary, circulatory, respiratory, neurological, psychiatric, digestive, endocrine, or other systemic diseases. 3) Concurrent rheumatoid arthritis, systemic lupus erythematosus, systemic sclerosis, relapsing polychondritis, or other autoimmune diseases. 4) Having malignant tumors or a history of malignant tumors. 5) Having contraindications for MRI examinations. 6) Suffering from diseases that the research team believes may interfere with the subject's treatment or compliance. 7) Participating in other related studies and receiving other osteoarthritis treatment regimens during this study. 8) Taking other medications that may affect the efficacy of osteoarthritis treatment during the treatment period. 9) Having a history of alcoholism or intravenous drug addiction within one year prior to enrollment. 10) Refusing to join the research team or refusing to sign the informed consent form.


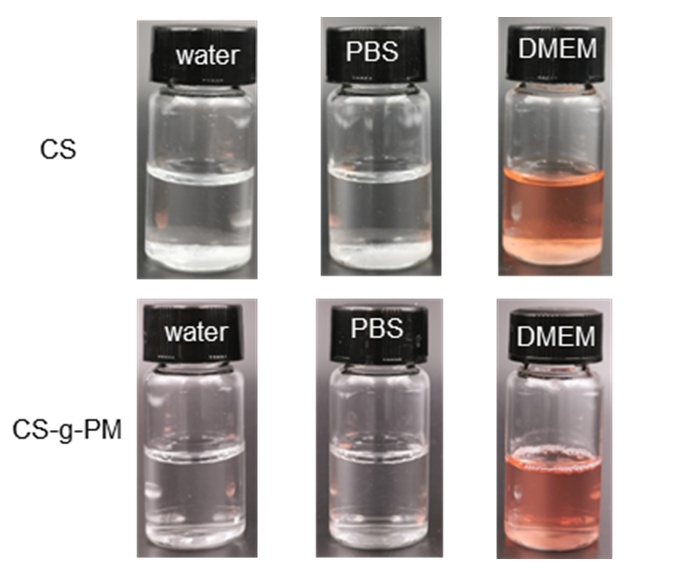


Fig S1 Physical diagram of the solubility of CS and CS-g-PM in deionized water, PBS and DMEM.


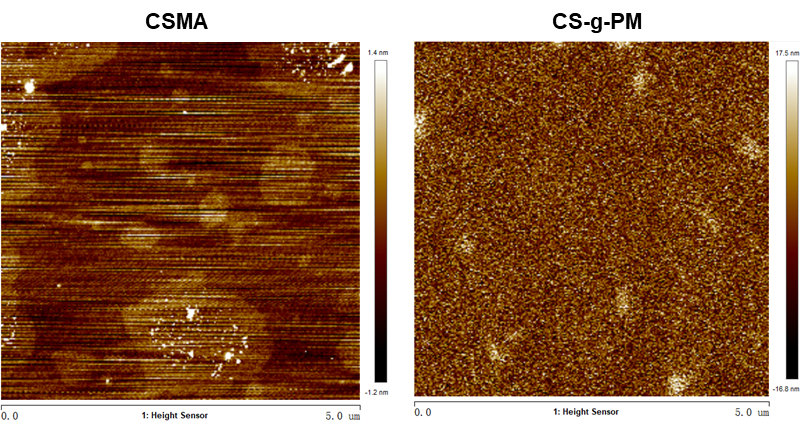


Fig. S2 AFM images showing the CSMA and CS-g-PM structures.


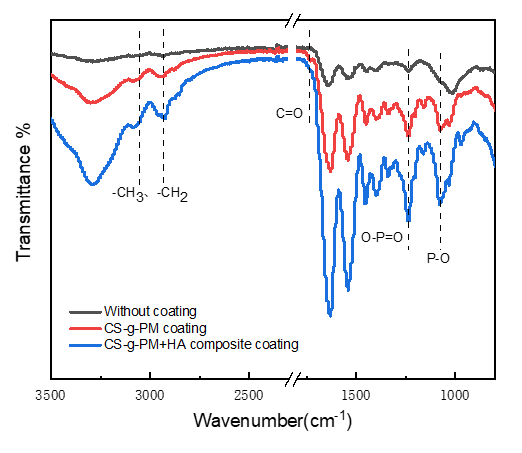


Fig. S3 FTIR spectra of without coating, CS-g-PM coating, and CS-g-PM+HA composite coating.


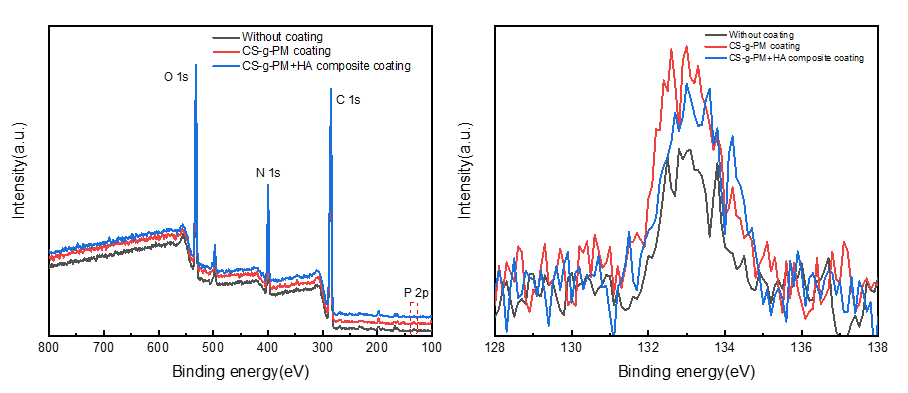


Fig. S4 XPS spectra of without coating, CS-g-PM coating, and CS-g-PM+HA composite coating.


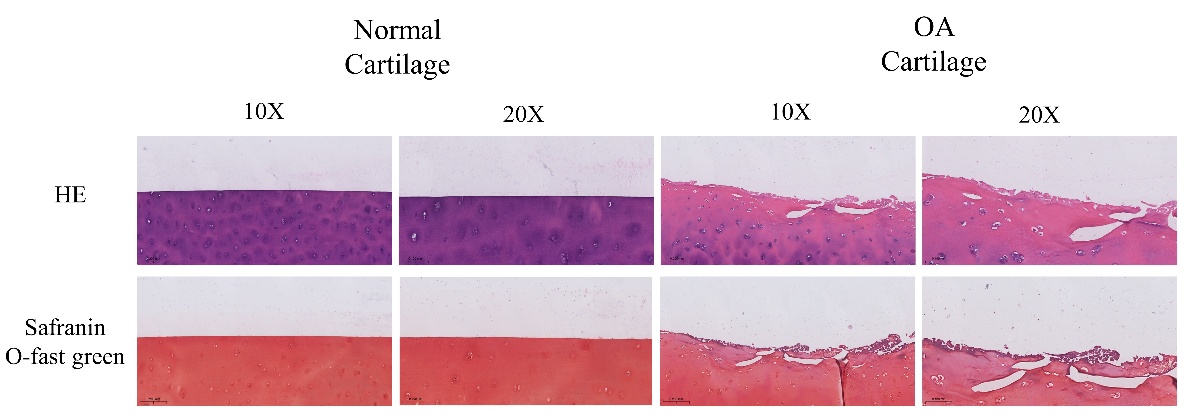


Fig. S5 HE staining and Safranin O-fast green staining from normal and OA human cartilage.


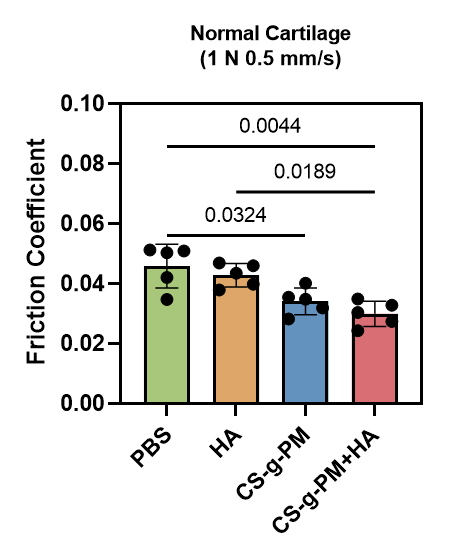


Fig. S6 The lubrication properties of the normal human cartilage at sliding velocities 0.5 mm/s under loads 1 N.


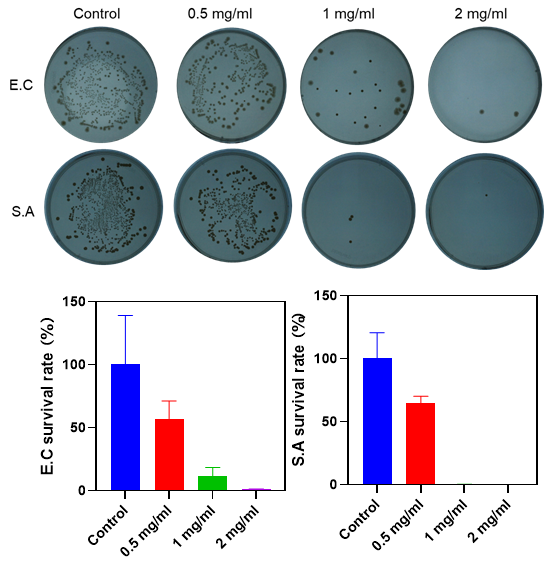


Fig. S7 Images of CS-g-PM with different concentrations against Escherichia coli and Staphylococcus aureus. and the survival rate of Escherichia coli or Staphylococcus aureus of the co-culture solution.


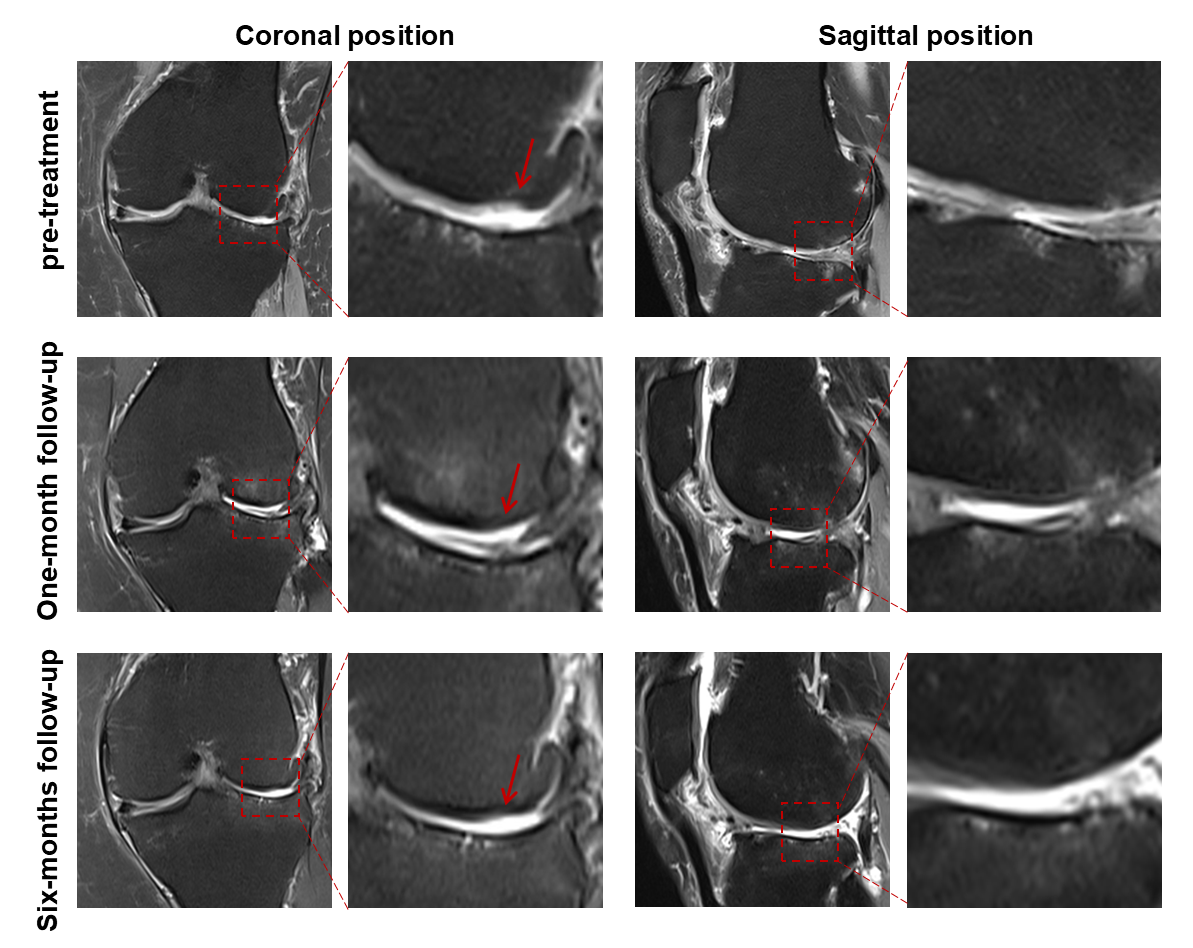


Fig. S8 Comparison of MRI for pre- and post-treatment for Case 1 in clinical trials.

Tab. S1 XPS atomic ratios of cartilage with or without coating.

| Sample | P2p | C1s | O1s | C/O |
| --- | --- | --- | --- | --- |
| Without coating | 0.25 | 42.59 | 40.56 | 1.05 |
| CS-g-PM coating | 0.37 | 47.56 | 36.59 | 1.30 |
| CS-g-PM+HA  composite coating | 0.32 | 47.45 | 36.88 | 1.28 |

Tab. S2 Results of in vitro biological tests of CS-g-PM

| Biological Test | Test Result |
| --- | --- |
| Hemolysis Test-Direct Contact | No Hemolysis |
| Cytotoxicity Test | No Cytotoxicity |
| Skin Sensitivity Test | No Sensitivity |
| Animal Intradermal Reaction Test | No Intradermal Reaction |
| Acute Systemic Toxicity Test | No Toxicity |
